# A non-classical PUF family protein in oomycetes functions as a pre-rRNA processing regulator and a target for RNAi-based disease control

**DOI:** 10.1371/journal.ppat.1013379

**Published:** 2025-07-31

**Authors:** Hui Feng, Tianli Liu, Chuanxu Wan, Zhichao Zhang, Yuanchao Wang, Xiaobo Zheng, Jie Wang, Wenwu Ye

**Affiliations:** 1 Tobacco Research Institute, Chinese Academy of Agricultural Sciences, Qingdao, Shandong, China; 2 Department of Plant Pathology, Nanjing Agricultural University, Nanjing, Jiangsu, China; 3 Key Laboratory of Soybean Disease and Pest Control (Ministry of Agriculture and Rural Affairs), Nanjing Agricultural University, Nanjing, Jiangsu, China; University of Cambridge, UNITED KINGDOM OF GREAT BRITAIN AND NORTHERN IRELAND

## Abstract

Ribosome biogenesis is an essential and tightly regulated process linked to cell proliferation and growth. However, its regulatory mechanisms in oomycetes, a group of organisms with significant agricultural and ecological importance, remain unclear. In this study, we identify Puf4, a non-classical PUF (Pumilio and FBF) family RNA-binding protein that plays a conserved and crucial role in pre-rRNA processing in oomycetes. Knockout of *PuPuf4* in *Pythium ultimum* or its ortholog *PsPuf4* in *Phytophthora sojae* results in defective vegetative growth, impaired development, and reduced pathogenicity. Specifically, PuPuf4 binds to the H68 component of 25S rRNA, and its knockout leads to overaccumulation of rRNA processing intermediates, including 5′ETS, ITS1, and ITS2 precursors. Additionally, the AG-rich motif identified as the first binding motif of L-shaped PUF proteins, including PuPuf4, APUM24, and ScPuf6, may contribute to their specific RNA-binding affinity due to its unique structural features. Given the conserved role of *Puf4* in oomycete pathogenicity, we developed the first nano-material-free dsRNA delivery system via zoospore-specific uptake, effectively attenuating virulence in *Pythium aphanidermatum* and *Ph. sojae* through RNAi targeting *Puf4*. This study presents novel findings on structural and functional conservation of Puf4 and offers a promising RNAi-based strategy for controlling oomycete plant diseases.

## Author summary

Ribosome biogenesis plays a crucial role in cell growth and proliferation. However, the regulation of this process in oomycetes, an ecologically and agriculturally important group of organisms, remains largely unknown. In this study, we identified Puf4, a non-classical PUF family RNA-binding protein of oomycetes, as a key factor promoting ribosome assembly in these pathogenic organisms. When we removed *Puf4* from two harmful oomycetes, *Pythium ultimum* and *Phytophthora sojae*, their growth was inhibited, and their ability to infect plants was diminished. Specifically, PuPuf4 binds to the H68 component of 25S rRNA and regulates the degradation of rRNA maturation byproducts. Additionally, we discovered a novel AG-rich motif that serves as a binding site for L-shaped PUF proteins. Furthermore, we developed a nano-material-free dsRNA delivery system targeting *Puf4*, which effectively reduced the virulence of *Pythium* and *Phytophthora* pathogens. Our findings not only uncover a fundamental process in oomycetes but also provide a sustainable approach to protect crops from these destructive microbes.

## Introduction

Post-transcriptional regulation of gene expression plays a crucial role in the growth and development of organisms. Post-transcriptional regulation encompasses processes including RNA splicing, editing, capping, polyadenylation, RNA transport, stability, and translation [1]. Most of these processes are regulated either directly or indirectly by RNA-binding proteins [2]. The PUF (Pumilio and FBF) family is a group of highly conserved eukaryotic RNA-binding proteins characterized by Pumilio homology domains [3]. PUF regulation of molecular events leads to cellular outcomes that are essential to maintenance of stem cell identity, mitochondrial biogenesis, and various aspects of organismal development [4]. The PUF family consists of at least three subfamilies. The classical PUF protein, consisting of eight helical tandem Pumilio repeats, binds to its targeted transcripts in a modular fashion, wherein contact between the nucleotides of the transcript and Pumilio repeat occurs in a one-to-one manner. The transcripts targeted by classical PUF proteins generally possess a core sequence motif (UGUANAUA) in their 3′ untranslated regions (UTRs). PuM90, a PUF protein of *P*. *ultimum*, specifically binds to a UGUACAUA motif in the mRNA 3′ UTR of PuFLP, thereby downregulating PuFLP expression to facilitate oospore formation [5]. Members of the Nop9 subfamily have C-shaped Pumilio isoforms that recognize sequence and structural elements of the target RNA comprising single-strand and duplex (stem-loop) conformations. Lastly, the subfamily of Puf-A in humans and ScPuf6 in *Saccharomyces cerevisiae* exhibits eleven Pumilio repeats arranged in an L-shaped pattern and binds to single- and double-stranded RNA or DNA with no apparent sequence specificity via interactions with the phosphate backbone [6]. No binding motifs have been identified in L-type PUF proteins.

The number of PUF family proteins varies among species. The genome of *Saccharomyces cerevisiae* encodes six PUF family members, while *Arabidopsis thaliana* contains 26 members [6]. The human genome is reported to encode one PUF protein with two isoforms, PUM1 and PUM2 [7]. Given the significance of PUF proteins for various *in vivo* functions, investigation and comparison of their biological roles and regulatory mechanisms across different species is of interest. Oomycetes, classified as stramenopile eukaryotes, are unicellular protists that physically resemble filamentous fungi; however, they are phylogenetically distinct and possess unique biological, genetic, and physiological characteristics [8,9]. As one of the most destructive oomycete pathogens, *Pythium ultimum* displays a wide host range and has been recorded on hundreds of plant species worldwide, causing damping-off and root rot, which lead to serious yield losses [10,11]. However, little is known about the molecular features underlying its disease cycle due to the lack of applicable gene editing technologies. In previous research, we successfully developed a clustered regularly interspaced short palindromic repeats (CRISPR)/Cas9 system-mediated gene knockout and conducted *in situ* complementation in *P*. *ultimum*, providing a platform for study of gene function in *Pythium* [5]. Despite investigation of eukaryotic functions and the binding mechanisms of classical PUF proteins, the evolution and biological functions of PUF proteins in oomycetes remain largely unclear.

The ribosome is one of the most abundant RNA–protein complexes, formed through incorporation of ribosomal proteins (r-proteins) into dynamically folding ribosomal RNA (rRNA) [12]. Ribosome biogenesis involves multiple processes, including pre-rRNA synthesis, ribosomal protein and ribosome biogenesis factor production, assembly of those components, and pre-rRNA processing. These processes must be regulated in coordination. Ribosome biogenesis begins with the biosynthesis of 45S pre-rRNA by RNA polymerase I in the nucleolus. This long transcript is processed into 18S, 5.8S, and 25S rRNA via the truncation of 5′ and 3′ external transcribed spacers (5′ETS and 3′ETS) and the removal of internal transcribed spacers 1 and 2 (ITS1 and ITS2) [13]. The ribosome is essential for translation, and thus critical to maintaining cellular vitality [14]. Previous studies have shown that PUF proteins are involved in ribosome biogenesis as ribosome processing factors. Specifically, *ScPuf6* plays a role in 60S biogenesis. In cells with *ScPuf6* deletion, pre-rRNA processing and 60S export are impaired, leading to under-accumulation of 60S subunits [15]. *APUM24* of *Arabidopsis thaliana* is an essential gene encoding a pre-rRNA processing-associated factor necessary for the removal of ITS2. In the absence of *APUM24*, embryos fail to develop and accumulate uridylated 27SB as well as 3′-extended 5.8S pre-rRNA, including polyadenylated byproducts. APUM24 binds to the 5.8S and ITS2 regions in a sequence-independent manner [16,17]. APUM23 is a nucleolar protein required for rRNA processing. An *APUM23* mutant accumulates 35S pre-rRNA, unprocessed 18S rRNA, and polyadenylated 5.8S pre-rRNA, indicating that APUM23 is involved in the degradation of rRNA maturation byproducts [18,19]. Despite reports highlighting a link between PUF proteins and ribosome biogenesis, the mechanisms through which PUF proteins function and are regulated in oomycetes remain largely unknown.

RNA interference (RNAi) is a conserved cellular defense process mediated by double-stranded RNA (dsRNA). This process regulates gene expression through chromatin modulation or degradation/inhibition of target mRNA, designated transcriptional gene silencing and post-transcriptional gene silencing, respectively [20,21]. Crops can be directly sprayed with dsRNA (spray-induced gene silencing, SIGS) targeting key genes of plant pathogens to induce specific silencing, providing an opportunity for sustainable eco-friendly disease management [22]. Although fungi such as *Botrytis cinerea* can efficiently absorb dsRNA from the environment [23]. Oomycetes such as *Phytophthora infestans* and *Ph*. *sojae* do not readily take up environmental dsRNA [24,25], possibly due to characteristics of their cell wall organization. In oomycetes, cellulose predominates the cell wall, whereas chitin is the dominant component of fungal cell walls [26]. Recent studies indicate that nanoparticles can overcome the cell barrier of *Phytophthora*, enhancing the efficiency of dsRNA uptake by cells. Star polycation (SPc) [27] and functionalized carbon dots [28] have successfully addressed the dsRNA delivery bottleneck for *Ph*. *infestans*, facilitating its efficient intracellular delivery. However, the use of nanomaterials presents several challenges, including concerns related to biosafety and environmental protection. The weak internalization of external dsRNA largely restricts the application of SIGS to pathogen control and gene function analyses [29]. Alternative methods to enhance the uptake of dsRNA by oomycetes and thus achieve the application of SIGS to oomycetes without the use of nanomaterials remain a critical issue to be addressed.

PuPuf4 contains varied Pumilio domain sequences and belongs to the non-classical PUF proteins. Analysis of its structure reveals a novel protein folding pattern with 11 Pumilio repeats in an L-like shape, despite having only six predicted Pumilio repeats based on its amino acid sequence. In this study, we demonstrate that PuPuf4 is a nuclear protein essential to oomycete pathogenicity. In the absence of *PuPuf4*, *P*. *ultimum* displays severe defects in vegetative growth, development and pathogenicity, and accumulates rRNA processing intermediates, including 5′ETS, ITS1, and ITS2 precursors. PuPuf4 binds to the H68 component of 25S rRNA in a sequence-independent manner. A novel AG-rich motif bound by PuPuf4 and other L-shaped PUF proteins has been identified. Exogenous application of *Puf4* dsRNA significantly reduced the pathogenicity of *Pythium* and *Phytophthora,* indicating Puf4 could serve as an excellent RNAi target for control of these oomycete pathogens. Hence, the present study reveals a new mechanism through which a PUF protein regulates rRNA to modulate growth and virulence and describes a new disease control technology developed based on this gene.

## Results

### PuPuf4 is an L-shaped PUF-like protein with 11 Pumilio repeats

**Fig 1:**
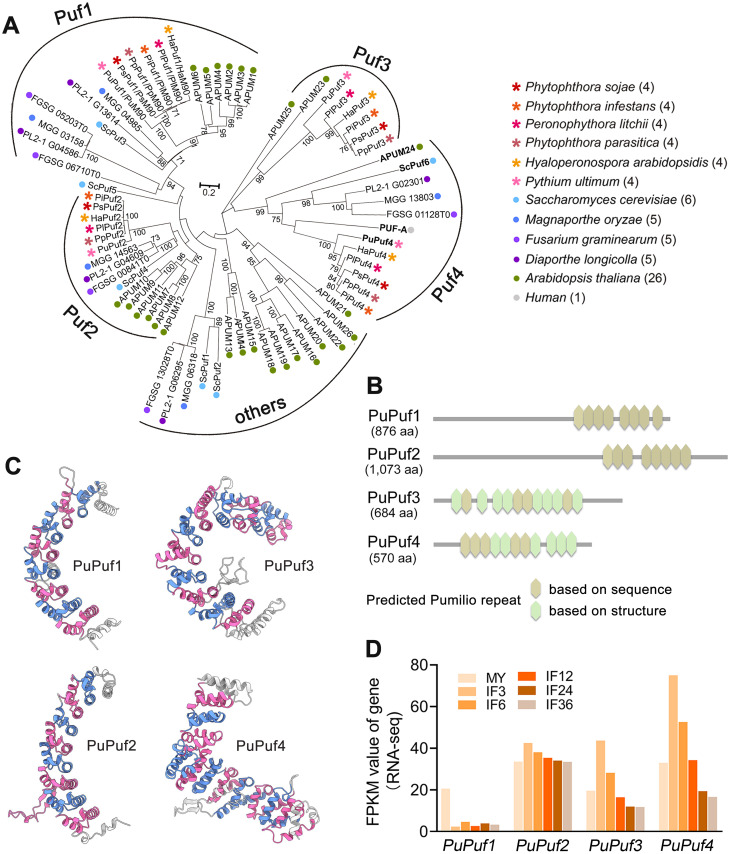
Characteristics of the PUF family. **(A)** Maximum-likelihood phylogenetic tree for PUF proteins in oomycetes, fungi, *Arabidopsis thaliana*, and humans. **(B)** Domain architectures of four *P*. *ultimum* PUF proteins predicted using SMART analysis based on their amino acid sequences (shown in yellow). The missing Pumilio repeat (highlighted in green) was computationally modeled to complete the characteristic structural framework depicted in **(C)** Ribbon diagrams showing the structural features of the PUF proteins PuPuf1 (classical), PuPuf2 (classical), PuPuf3 (C-shaped), and PuPuf4 (L-shaped). http://smart.embl-heidelberg.de/. **(D)** RNA-seq analysis was performed to quantify the transcript levels of *Puf* genes during the infection of soybean hypocotyls at 3, 6, 12, 24, and 36 hours post-inoculation (hpi). MY represents the mycelium control, while IF3, IF6, IF12, IF24, and IF36 denote the samples collected at 3, 6, 12, 24, and 36 hpi, respectively.

PUF proteins are conserved among species, with varying quantities found in *Arabidopsis*, fungi, and humans (Fig 1A, S1 Table). Manual annotation has indicated that representative oomycete species in the genera *Phytophthora*, *Peronospora*, and *Pythium* typically possess four PUF proteins. *Arabidopsis thaliana* encodes 26 PUF proteins, fungi generally contain five PUF proteins, yeast has six PUF proteins, and the human genome encodes a single PUF protein (Fig 1A). Phylogenetic analysis of 72 PUF proteins across 12 species revealed that these proteins can be categorized into five distinct groups. Domain prediction based on amino acid sequences revealed that PuPuf1 and PuPuf2 each contain eight Pumilio repeats, while PuPuf3 contains four and PuPuf4 contains five (Fig 1B). Subsequent protein structure analysis using AlphaFold3 revealed that PuPuf1 and PuPuf2, each containing eight repeat motifs, form a characteristic meniscus-shaped structure, indicating they are classical PUF proteins. In contrast, PuPuf3, which has eleven repeat motifs, adopts a C-shaped structure while PuPuf4 forms an L-shaped structure (Fig 1C). PuPuf4 also has eleven repeat motifs, despite only five repeats predicted based on its amino acid sequence (Fig 1B), indicating the Pumilio repeats of PuPuf4 cannot be fully predicted through amino acid sequence analysis. Based on RNA sequencing (RNA-seq) analysis of *Pythium ultimum*-inoculated soybean hypocotyls at 3, 6, 12, 24, and 36 hours post-inoculation (hpi), we observed that the transcript level of PuPuf4 was significantly upregulated at 3 hpi (2.2-fold increase compared to the mycelium control [MY]; Fig 1D). The early-stage elevation of *PuPuf4* transcripts during infection further suggests that *PuPuf4* plays a critical role in the infection process.

Phylogenetic analysis positions PuPuf4 within a distinct clade of non-classical PUF proteins, with close relationships to APUM24, ScPuf6, and PUF-A (Fig 1A). Unlike classical PUF proteins, these homologs share an L-shaped architecture composed of 11 Pumilio repeats (S1A Fig). This unique conformation arises from two modular subdomains. The C-terminal subdomain (residues 206–546) contains eight canonical Pumilio repeats (C-R1-C-R8) and a C-terminal pseudorepeat (C-R8′); this region adopts a curved conformation structurally analogous to classical PUF proteins such as PuM90. However, two repeats (C-R1 and C-R5) exhibit divergent structural features (S1B Fig). The N-terminal subdomain (residues 98–201) contains three Pumilio repeats (N-R1-N-R3) flanked by an N-terminal pseudorepeat (N-R1′), forming an arm perpendicular to the C-terminal subdomain. Classical PUF proteins (e.g., PuM90) employ conserved α2 helix motifs in their repeats that recognize RNA via sequence-specific interactions, typically binding to UGUA [A/U/C]AUA motifs (S2A and S2B Fig) [5]. While the C-terminal domain of PuPuf4 exhibits structural similarity to PuM90, critical RNA-binding residues are not conserved (S2D Fig) and structural deviations in C-R1 and C-R5 disrupt the canonical RNA-binding interface (S1B and S2C Figs). These observations suggest that PuPuf4 may employ an alternative RNA-binding mechanism or lack RNA-binding activity altogether. Comparative analysis of the oomycete homologs PsPuf4 (*Phytophthora sojae*, 7 repeats) and PaPuf4 (*Pythium aphanidermatum*, 6 repeats) reveals conservation of the L-shaped architecture despite variability in repeat number (S3A and S3B Fig). The structural conservation of Puf4 across oomycete species underscores its evolutionary and biological significance as a non-classical PUF protein with specialized functional roles.

### PuPuf4 is essential to vegetative growth and pathogenicity in *P*. *ultimum*

To investigate the biological functions of *PuPuf4*, we employed a CRISPR/Cas9-mediated gene replacement strategy using the *hph* gene, which encodes hygromycin B phosphotransferase, for knockout of *PuPuf*4. Additionally, an in situ gene complementation assay was conducted as previously described [5]. Through these strategies, we obtained both *PuPuf4* knockout mutants and complemented mutants, and their phenotypes were subsequently analyzed. The results obtained from two representative *PuPuf4* knockout mutants (ΔPuPuf4-1/2) and two representative *PuPuf4* complemented strains (ΔPuPuf4-C1/2) are presented. The wild-type strain (WT) and two empty-vector lines (EV, in which *PuPuf4* knockout was unsuccessful, for comparison with the WT; and ΔPuPuf4-EV, in which *PuPuf4-m* was not successfully complemented, for comparison with ΔPuPuf4-1) were included as controls (S4 Fig).

**Fig 2:**
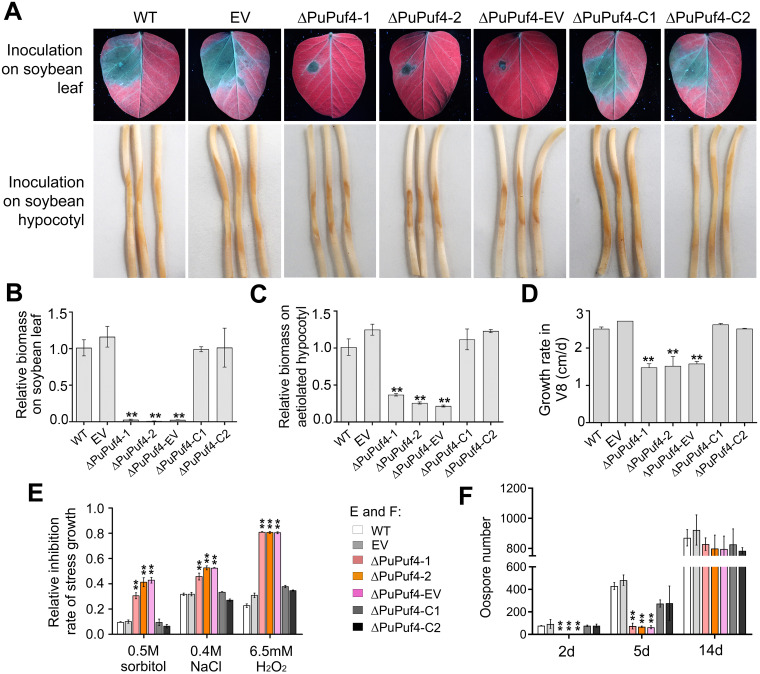
ΔPuPuf4 mutants are defective in virulence and growth. **(A)** Infection lesions on soybean leaves at 48 hpi and soybean hypocotyls at 24 hpi. **(B)**, **(C)** Relative *P*. *ultimum* biomass detected through qRT-PCR at 48 h after leaf infection (B) and 24 h after hypocotyl infection (C). **(D)** Growth rates on V8 medium. **(E)** Relative inhibition rate on V8 medium containing 0.5M sorbitol, 0.4M NaCl, and 6.5 mM H_2_O_2._ **(F)** Oospore number after culturing for 2 d, 5 d, and 14 d. Asterisks indicate significant differences relative to the WT at P < 0.05 (*) or P < 0.01 (**).

When cultured on nutrient-rich V8 medium, clear reductions in growth were observed for ΔPuPuf4 and ΔPuPuf4-EV (S5 Fig). The average growth rate among WT, EV, and ΔPuPuf4-C colonies was approximately 3.04 cm/day. Under the same conditions, the growth rate of ΔPuPuf4 mutants and ∆PuPuf4-EV was only 1.88 cm/day (Fig 2D). To explore the role of *PuPuf4* in the responses to abiotic stresses including osmotic, salt, and oxidative stress, we cultured all strains in medium containing 0.5 M sorbitol, 0.4 M NaCl, and 6.5 mM H_2_O_2_, and then analyzed mycelium inhibition rates. The results showed that ΔPuPuf4 and ΔPuPuf4-EV exhibited significantly higher inhibition rates than WT, EV, and ΔPuPuf4-C (P < 0.01) when exposed to sorbitol, NaCl, and H_2_O_2_ (Figs 2E and S5). Previous studies have shown that *ScPuf6*, a low temperature-induced factor, is required for 60S pre-ribosome export at low temperature, and that growth of the ΔScPuf6 mutant is impaired at 20 and 25°C [30]. In accordance with results for *ScPuf6*, we found that the growth of the ΔPuPuf4 mutants, determined from the size of colonies, showed more severe reduction at 20 and 15°C compared with the WT (S6 Fig). These results demonstrate that knockout mutants are more sensitive to low temperature than the WT.

With respect to oospore formation, WT, EV, and ΔPuPuf4-C began to form oogonia after two days of cultivation. Oogonia of ΔPuPuf4 and ΔPuPuf4-EV appeared on day 5 and a significant reduction (83%) in the number of oogonia was observed. However, no significant difference in oogonia number between WT and ΔPuPuf4 was present after 14 days of culture growth (Figs 2F and S7). No difference in oogonia morphology was observed among strains (S7 Fig). These findings suggest that *PuPuf4* delays oospore formation, potentially due to slowed mycelial growth.

To determine the effect of *PuPuf4* deletion on *P*. *ultimum* infection, mycelial plugs of all strains were inoculated onto soybean plants (Hefeng 47 variety). After inoculation on soybean leaves for 48 h, disease spots were observed under ultraviolet light. Soybean leaves inoculated with WT, EV, and ΔPuPuf4-C showed disease spots covering large areas, while ΔPuPuf4 and ΔPuPuf4-EV showed smaller disease spots with no expansion (Fig 2A). Measurement of relative *P*. *ultimum* biomass in infected soybean leaves revealed little pathogen biomass in the ΔPuPuf4 and ΔPuPuf4-EV-infected tissues, with less than 5% of the levels found in WT-, EV-, and ΔPuPuf4-C-infected leaves (Fig 2B). The infection assay was also performed on hypocotyls. At 24 h post-inoculation (hpi), the WT, EV, and ΔPuPuf4-C strains produced typical water-soaked decay around the disease lesions (Fig 2A), whereas the lesion areas of ΔPuPuf4 and ΔPuPuf4-EV were significantly smaller and pathogen biomass was reduced by 60% compared to WT and EV (Fig 2C). Taken together, these results indicate that PuPuf4 is a critical pathogenic factor in *P*. *ultimum*.

### PuPuf4 is involved in 45S pre-rRNA processing

**Fig 3:**
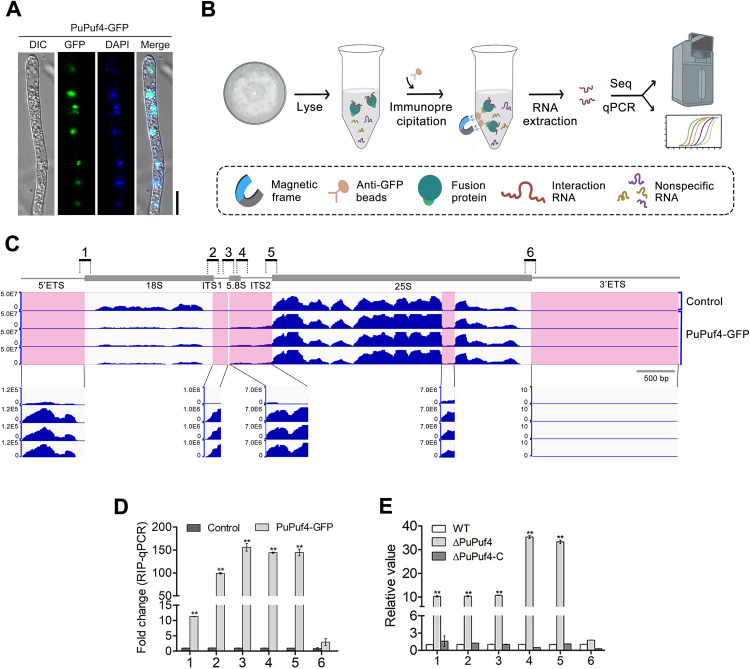
PuPuf4 is involved in 45S pre-rRNA processing. **(A)** Subcellular localization of PuPuf4-GFP. DAPI (4′,6-diamidino-2-phenylindole) staining was performed by adding DAPI to the cultures 5 min prior to microscopic analysis. DIC: differential interference contrast; Merge: overlay of DIC, GFP fluorescence and DAPI staining. Bar, 10 μm. **(B)** Flow chart of RIP-seq. *P*. *ultimum* culture is lysed, incubated with anti-GFP beads, and then enriched with the protein–RNA mixture using a magnetic rack. Complexes are removed from the beads and then RNA is eluted and prepared for sequencing and qPCR. **(C)** Integrative Genomics Viewer results showing PuPuf4 binding peaks on rRNA precursors. The control represents the RNA segment where GFP binds. In comparison to GFP, PuPuf exhibits binding peaks in specific regions, including the 5′ETS, ITS1, 5.8S, ITS2, and 25S regions. (D) qRT-PCR analysis of RNA coimmunoprecipitated with PuPuf4-GFP protein with anti-GFP antibodies. Enrichment of pre-rRNA processing intermediates containing specific regions (regions 1 to 6) through co-IP was calculated using values obtained from the PuPuf4-GFP sample compared to those from the GFP sample. Error bars represent standard deviation (n = 3). Asterisks indicate statistically significant differences between values obtained with GFP (control) and the WT expressing PuPuf4-GFP based on Student’s t-test (**P < 0.01). **(E)** Relative levels of processing intermediates in the WT (wild type), ΔPuPuf4 (knockout mutant), and ΔPuPuf4-C (complemented line). Asterisks indicate statistically significant differences between the mutants and WT based on Student’s t-test (**P < 0.01).

To investigate the subcellular localization of PuPuf4, a construct carrying green fluorescent protein (GFP)-tagged PuPuf4 in the pTOR vector was transformed into *P*. *ultimum*. Following treatment with DAPI (4′,6-diamidino-2-phenylindole), nuclei of PuPuf4-GFP transformants were stained blue and distributed as dots. Green fluorescence was also distributed in dots, and the two fluorescence signals show overlap (Fig 3A). This pattern indicates that the PuPuf4 protein accumulates in the nucleus. As PuPuf4 contains an RNA-binding domain, Pumilio [3], we investigated whether PuPuf4 regulates RNA through direct binding to its target RNAs. To test this possibility, we performed the RNA immunoprecipitation (RIP) assay followed by sequencing (RIP-seq) (Fig 3B) using PuPuf4-GFP and GFP, a strain expressing only GFP, which was used as a control (S8 Fig). Using RIP-seq, we identified 432 PuPuf4 binding peaks (P < 0.0005, fold change > 1.5), of which 272 peaks corresponded to 219 protein-coding genes (S2 Table). We analyzed the distribution of these peaks among protein-coding transcripts. The results showed that in addition to the binding peaks for mRNA, binding peaks for rRNA were also present. Integrative Genomics Viewer visualization showed that PuPuf4 had significant binding peaks in the 5′ETS, ITS1, 5.8S, ITS2, and partial region of 25S compared to the control (Fig 3C). We quantified the levels of rRNA that coimmunoprecipitated with PuPuf4 by performing quantitative reverse transcription polymerase chain reaction (qRT-PCR) with several pairs of primers to amplify different regions of pre-rRNA (Fig 3C). Among the various fragments produced from the pre-rRNA, fragments containing 5′ETS, ITS1, and ITS2 (primer sets 1, 2, 3, 4, and 5) were significantly enriched, depending on the expression of GFP-tagged PuPuf4 (Fig 3D). These results suggest that PuPuf4 specifically binds to pre-rRNA intermediates containing 5′ETS, ITS1, and ITS2 sequences.

External and internal transcriptional spacers (5′ETS, 3′ETS, ITS1, and ITS2) in the pre-rRNA are removed by endonucleases and exonucleases. During the cutting processes of various nucleases, a number of pre-rRNA processing intermediates are generated, which are rapidly degraded as non-functional byproducts [17]. To investigate whether excessive accumulation of pre-rRNA processing products occurs in the ∆PuPuf4 mutant, we monitored the rRNA processing steps through qRT-PCR to detect and quantify processing intermediates. Therefore, as shown in Fig 3C, six pairs of qRT-PCR primers were designed. Intermediates containing 5′ETS, ITS1, and ITS2 sequences accumulated in excess in the PuPuf4 mutant, while 3′ETS processing intermediate amounts in the ΔPuPuf4 mutant were similar to those of WT (Fig 3E).

To identify other ribosome processing factors that interact with Puf4, we obtained a *P*. *ultimum* transformant expressing GFP-tagged PuPuf4 and performed protein co-immunoprecipitation (co-IP) assays on lysates of this transformant using a GFP antibody as bait. Through analysis of the complex that co-precipitated with PuPuf4 via mass spectrometry (MS), we identified the K-loop GTPase Nog2 (PYU1_T009523), which was present in all three MS experiments (S3 Table). Nog2, which anchors both the pre- and post-rotation states of the 5S ribosomal ribonucleoprotein (Nog2pre and Nog2post), is an essential ribosome biogenesis factor that engages the rRNA A-loop during sequential steps of 60S maturation [31,32]. and we therefore named this protein PuNog2. To verify the protein–protein interaction between PuPuf4 and PuNog2, PuPuf4-GFP was co-expressed with PuNog2-FLAG in *P*. *ultimum* (S9 Fig) and lysates of the mycelium were subjected to co-IP analysis. The co-IP assay showed that PuPuf4-GFP interacts with PuNog2-FLAG (S10A Fig). To confirm the interaction between the two proteins *in vitro*, we employed the yeast two-hybrid assay. Transformants expressing the AD-PuNog2 and BD-PuPuf4 constructs (constructed with the vectors pGADT7 and pGBKT7, respectively) showed no β-galactosidase activity on synthetic defined (SD)-Leu-Trp-His-Ade plates, indicating that PuPuf4 does not directly interact with PuNog2 (S10B Fig). To investigate whether PuNog2 is involved in rRNA processing, gene knockout of PuNog2 was performed using the method described above; after many attempts, no knockout mutant was obtained, suggesting that PuNog2 is essential and the deletion of its encoding gene leads to the death of *P*. *ultimum*.

### PuPuf4 binds to H68 rRNA within a 60S pre-ribosome

**Fig 4:**
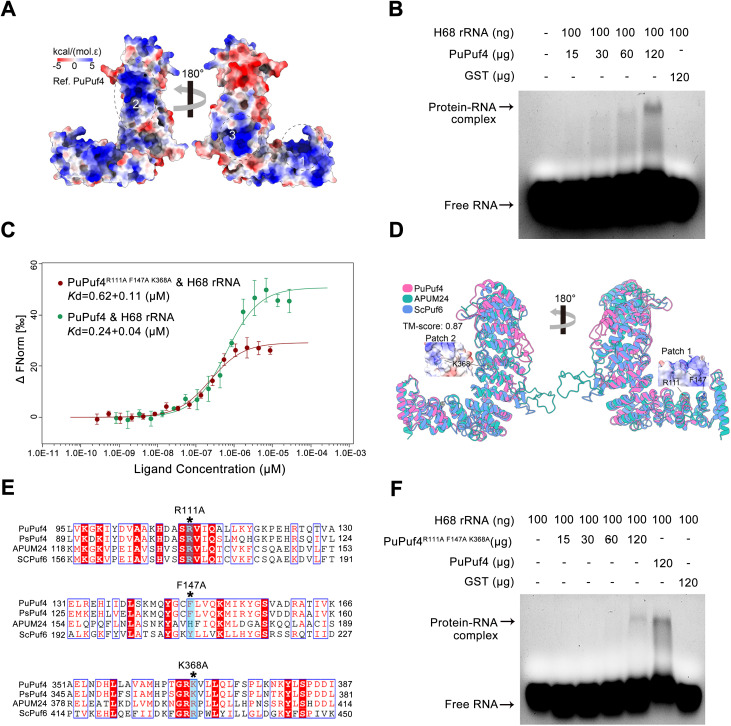
PuPuf4 binds H68 rRNA. **(A)** Electrostatic surface representation of the structure of PuPuf4. Three distinct basic patches are indicated with dotted ovals. **(B)** EMSA results showing that PuPuf4 bound H68 rRNA. **(C)** MST results showing that PuPuf4 bound H68 rRNA (*K*_d_ = 0.24 μM, green curve), while mutation of R111, F147, and K368 in PuPuf4 attenuated its binding to H68 rRNA (*K*_d_ = 0.62 μM, red curve). **(D)** Superposition of PuPuf4, APUM24, and ScPuf6. K368 is located on patch 2, and R111 and F147 are located on patch 1. **(E)** Partial sequence alignment of non-canonical PUF proteins including PuPuf4, PsPuf4, APUM24, and ScPuf6. The residues R111, F147, and K368, indicated by asterisks, were replaced with A. **(F)** PuPuf4^R111A, F147A, K368A^ nearly abolished detectable binding to H68 rRNA but formed a weak RNA–protein complex at high levels of PuPuf4^R111A, F147A, K368A^.

Previous studies have shown that ScPuf6 strongly interacts with helix 68 (H68) of 25S rRNA [30]. R172 and Y208 are components of the basic Patch 1 with positive electrostatic potential, which is critical to correct 7S pre-rRNA processing and localization of ASH1 mRNA [15]. The R431E channels of Patch2 affect yeast growth and RNA binding [30]. However, the RNA-binding site of PuPuf4 on the 60S pre-ribosome has remained unclear. The charge distributions of PuPuf4 and ScPuf6 are very similar (Figs 4A and S11). Three distinct patches of positive electrostatic potential on the surfaces of PuPuf4 and ScPuf6 appear well suited to interact with negatively charged nucleic acids (labeled 1, 2, and 3 in Fig 4A). We hypothesize that PuPuf4 exhibits an RNA-binding mode similar to that of Puf6. Using electrophoretic mobility shift assay (EMSA) and microscale thermophoresis (MST), we found that PuPuf4 can bind the H68 rRNA sequence (Fig 4B and 4C). The R172, Y208, and R431 residues of ScPuf6 correspond to R111, F147, and K368 in PuPuf4, among which K368 is located on Patch 2, and R111 and F147 are located on Patch 1 (Fig 4D and 4E). When R111, F147, and K368 were mutated, PuPuf4^R111A F147A K368A^ exhibited little detectable binding to H68. H68 formed a weak RNA–protein complex only at a high concentration of PuPuf4^R111A F147A K368A^ (Fig 4F). MST experiments revealed that PuPuf4 bound to the H68 rRNA sequence with dissociation constant (*K*_d_) = 0.24 + 0.04 μM. Mutation of R111, F147, and K368 in PuPuf4 attenuated its binding of H68 rRNA to *K*_d_* *= 0.62 + 0.11 μM (Fig 4C). These data suggest that PuPuf4 directly binds H68 and that R111, F147, and K368 are required for this interaction.

### PuPuf4 binds and regulates genes involved in ribosome biogenesis

**Fig 5:**
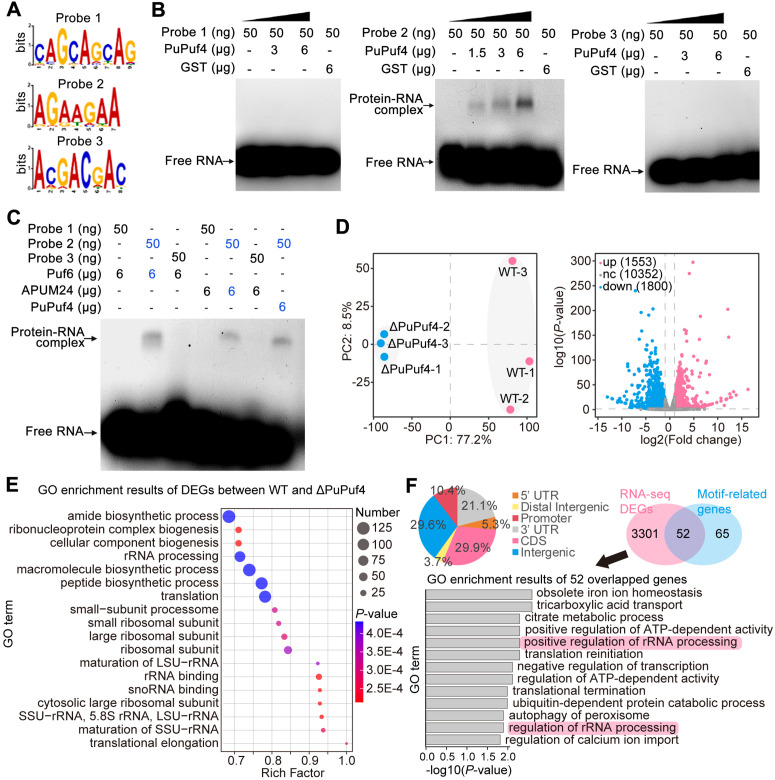
Genes associated with ribosome biogenesis are bound and regulated by PuPuf4. **(A)** Sequence of the enriched motif among the peak sequences of PuPuf4 target genes. **(B)** *In vitro* binding of PuPuf4 to three motifs in the EMSA assay. **(C)** EMSA assay showing that non-canonical PUF proteins including ScPuf6 and APUM24 bound AGAAGAA. **(D)** Principal component analysis of transcriptome data and overview of DEGs in the *PuPuf4* knockout compared to WT. **(E)** GO enrichment results of DEGs between WT and ΔPuPuf4. **(F)** Distribution of PuPuf4-bound peaks within protein-coding gene bodies divided into 5′-untranslated regions (UTRs), coding sequences (CDSs), 3′-UTRs, and intronic regions. Venn diagram showing the overlap of DEGs and motif-related genes with GO enrichment results of the overlapping genes.

To identify the PuPuf4-binding cis-element in the target mRNAs, 432 PuPuf4 binding peaks were analyzed using the Multiple Expectation maximizations for Motif Elicitation (MEME) suite. The three most significantly enriched RNA motifs (CAGCAGCAG, AGAAGAA, and ACGACGAC) were determined through MEME analysis (Fig 5A). To verify whether PuPuf4 can directly bind these three RNA motifs, 5-carboxy-fluorescein (FAM)-labeled probes were synthesized and employed to verify the interaction via EMSA. In the EMSAs, glutathione S-transferase (GST)-tagged PuPuf4 showed binding activity to probe2 but not probe1 or probe3 (Fig 5B). Previous studies have shown that ScPuf6/Puf-A binds its targets in a sequence-independent manner, and the binding motif of L-type PUF proteins has not yet been reported. To verify whether probe2 is a binding motif for L-type PUF proteins including ScPuf6 and APUM24, EMSA experiments were conducted for ScPuf6 and APUM24. As shown in Fig 5C, despite the relatively low protein sequence similarity of PuPuf4 to ScPuf6 and APUM24 (26.3% and 27.6%, respectively) (S12 Fig), ScPuf6 and APUM24 bound to probe2 but not to probe1 or probe3. These data demonstrate that AGAAGAA is a novel binding motif for L-type PUF proteins.

We performed RNA-seq on the WT and ΔPuPuf4 strain using three independent biological replicates. Compared to the WT, 1553 and 1800 genes were significantly upregulated and downregulated, respectively, in ΔPuPuf4 (Fig 5D and S4 Table). Gene Ontology (GO) results for differentially expressed genes (DEGs) between WT and ΔPuPuf4 showed that multiple pathways related to ribosome biogenesis exhibited significant enrichment (Fig 5E). We analyzed the distribution of these peaks within protein-coding transcripts and found that 29.9% of peaks reside in the coding sequences, 21.1% and 5.3% of peaks reside in the 3′- and 5′-UTRs, respectively, and the remaining peaks (29.6%) reside in intronic regions (Fig 5F). Sequence analysis demonstrated that peaks of 117 target genes contain the AGAAGAA motif (S2 Table). To explore the relationship between targets containing the AGAAGAA motif and DEGs of the WT *vs*. ΔPuPuf4, we overlaid the two groups and found that the overlapping genes showed significant enrichment in ribosome-associated genes, further suggesting that the binding of PuPuf4 to mRNAs is related to the ribosome (Fig 5F). Among the 52 differentially expressed genes (DEGs) containing the PuPuf4 binding motif, seven genes were up-regulated and 45 were down-regulated in the *PuPuf4* knockout mutant (S4 Table). This suggests that most genes bound to PuPuf4 have undergone degradation.

### Exogenous application of *Puf4*-dsRNA attenuates *Pythium* and *Phytophthora* pathogen infection

**Fig 6:**
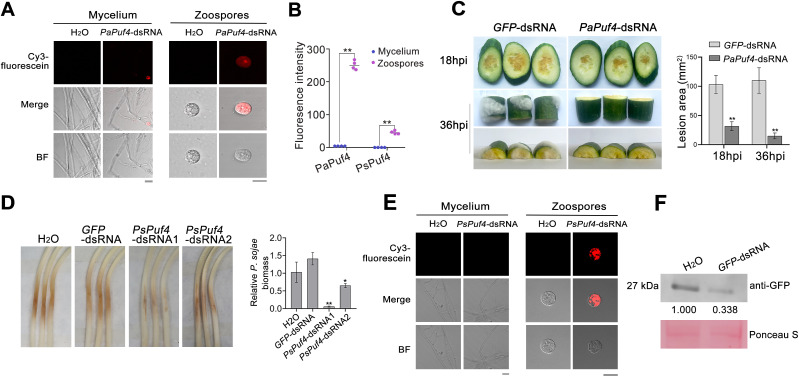
Application of exogenous *Puf4*-dsRNAs impairs the virulence of *Pythium aphanidermatum* and *Phytophthora sojae.* **(A)**, **(E)** Observation of dsRNA uptake efficiencies in mycelium and zoospores of *P*. *aphanidermatum* and *Ph*. *sojae*. Cy3-labeled dsRNA (500 bp, 150 ng μL^−1^) was added and micrococcal nuclease treatment was performed 30 min before image acquisition using a Leica SP8 confocal microscope with excitation at 555 nm. Scanning was performed with the filter set to 570 nm. Cy3 signals were detected inside the target cells. Pictures were taken at 12 h post-treatment. Bar, 10 μm. **(B)** Fluorescence intensity in mycelium and zoospores treated with dsRNA was quantified using ImageJ software. Four images of each treatment were analyzed. Asterisks (**) indicate statistical significance compared with mycelium (P < 0.01). **(C)** Lesions on cucumber slices or blocks inoculated with *P*. *aphanidermatum* zoospores. The lesion areas were measured with ImageJ. Asterisks indicate significant differences compared with *GFP*-dsRNA at P *<* 0.01 (**). **(D)** Etiolated soybean seedlings inoculated with *Ph*. *sojae* zoospore suspension. Pictures were taken at 48 hpi. Data were subjected to statistical analysis using two-tailed t-test. Asterisks indicate significant differences relative to H_2_O at P < 0.05 (*) or P *<* 0.01 (**). **(F)** The level of GFP protein was dramatically reduced in *Ph*. *sojae* treated with corresponding *GFP*-dsRNA. Etiolated soybean seedlings were inoculated with *Ph*. *sojae* (overexpressing GFP) following treatment with *GFP*-dsRNA. The relative protein level was detected through western blotting of total protein extracted from the pathosystem. After quantification using ImageJ, relative band intensity was calculated. Ponceau staining indicated that total protein contents were consistent among samples from different treatments.

While the application of dsRNA has been demonstrated in *Phytophthora*, its efficacy in *Pythium* remains to be elucidated. Additionally, the internalization of external dsRNA currently depends on nanomaterials for delivery, but this approach is associated with low uptake efficiency [24]. We hypothesize that the presence of a cell wall during the hyphal stage could hinder the uptake of dsRNA. Therefore, we propose that employing zoospores, which lack cell walls, may significantly enhance the uptake of dsRNA. However, *P*. *ultimum* rarely produces zoospores. Thus, *P*. *aphanidermatum*, a destructive oomycete pathogen that produces zoospores was selected as the test object. To explore whether *Puf4* can be used as a RNAi target for the control of *Pythium*, the *Puf4* gene of *P*. *aphanidermatum*, *PaPuf4*, was cloned and *PaPuf4*-dsRNA (225–724 bp) was generated under control of the double T7 promoter. Mycelium and cell wall-free zoospores of *P*. *aphanidermatum* were cultured with fluorescein-labeled *PaPuf4*-dsRNA for 24 hours followed by detection of fluorescence signals. Confocal microscopic analysis confirmed that dsRNA could not enter the mycelium but could enter zoospores of *P*. *aphanidermatum* (Fig 6A and 6B), indicating that the cell wall blocks entry of dsRNA. Cucumber samples were inoculated with the zoospores of *P*. *aphanidermatum* after treatment with dsRNA for 30 min. Cucumber inoculated with zoospores treated with dsRNA showed reduced disease symptoms and lesion sizes compared to controls treated with *GFP*-dsRNA (Fig 6C). Thus, externally applied *PaPuf4*-dsRNA reduced the virulence of *P*. *aphanidermatum*.

To determine whether dsRNA of *Puf4* has an effect on *Phytophthora*, the function of PsPuf4 was investigated. To this end, *PsPuf4* knockout mutants were generated using the strategy described by Fang [33]. The phenotypic characteristics of three representative *PsPuf4* knockout mutants (ΔPsPuf4-1/2/3) are presented (S13 Fig). ΔPsPuf4 mutants displayed approximately 35% reduction in growth rates compared to the WT (p < 0.01) and displayed higher inhibition rates than WT and EV in medium containing 1 M sorbitol, 0.6 M NaCl, and 5 mM H_2_O_2_ (S14 Fig). Together, this evidence indicates that *Puf4* mediates mycelial growth and the signaling pathway used to resist abiotic stresses in oomycetes. ΔPsPuf4 began to form oogonia at day 6, which was later than the WT at day 4, and ΔPsPuf4 had a significantly smaller oogonia number on day 6 than the WT. However, no significant difference in oogonia number between the WT and ΔPsPuf4 was present on day 14 (S15A Fig). No difference was apparent in oogonia morphology among strains (S15B Fig). These results indicate that *Puf4* delayed oospore formation, possibly by slowing the growth of mycelium. The tested strains (WT, EV, and ΔPsPuf4) were induced to produce zoospores and then inoculated onto plant hypocotyls, and similar results were obtained. At 48 hpi, soybean seedlings inoculated with ΔPsPuf4 developed only small necrotic lesions at the site of inoculation and showed a 90% reduction in oomycete biomass, whereas P6497 and EV produced normal disease lesions (S16A and S16B Fig).

To validate whether dsRNA of *PsPuf4* controls disease caused by *Phytophthora*, we generated constructs expressing *PsPuf4*-dsRNA1 (243–742 bp) and *PsPuf4*-dsRNA2 (959–1499 bp) under the double T7 promoter. Zoospores of *Ph***.** *sojae* were treated with *PsPuf4*-dsRNA1 and *PsPuf4*-dsRNA2 for 30 min and then inoculated on soybean hypocotyls for 48 h, followed by photography and sampling. As shown in Fig 6D, *PsPuf4*-dsRNA1 reduced the size of necrotic lesions at the site of inoculation and reduced biomass by more than 90%, indicating that *PsPuf4*-dsRNA1 is the more efficient dsRNA (Fig 6D). Confocal microscopy confirmed that the dsRNA could not enter the mycelium but could enter the zoospores of *Ph*. *sojae* (Fig 6B and 6E), indicating that the cell wall blocks entry of dsRNA.

To investigate how dsRNA reduces the pathogenicity of *Ph*. *sojae*, we examined its effect on gene expression. The transcription level of *PsPuf4* was measured using qPCR. Compared with the control (H_2_O or *GFP*-dsRNA), no significant change in mRNA levels occurred following treatment with *PsPuf4*-dsRNA (S17 Fig). This finding indicates that gene translation might be affected by PsPuf4. Therefore, *Ph*. *sojae* expressing GFP was used for the experiment. Following treatment of GFP-expressing *Ph*. *sojae* with GFP dsRNA, we detected the expression level of GFP protein via western blot analysis (Fig 6F). The results indicated that, compared to the water control, the expression level of GFP protein was reduced following *GFP*-dsRNA treatment. These findings suggest that the effect of dsRNA on *Ph*. *sojae* was induced by the inhibition of *PsPuf4* translation and not a transcriptional change.

## Discussion

The ribosome is required for translation, making it absolutely essential for maintaining the vitality of the organism. Therefore, defects in ribosome biogenesis or function have negative effects on all cellular functions [34]. More than 200 ribosome biogenesis factors are involved in the process of ribosome biogenesis [31]. In this study, we identified Puf4, a non-canonical PUF protein, as a key regulator of pre-rRNA processing with roles in the growth and virulence of oomycetes. Knockout of *PuPuf4* in *P*. *ultimum* led to significant accumulation of intermediates containing 5′ETS, ITS1, and ITS2, along with marked defects in growth and pathogenicity. A novel AG-rich binding motif has been identified for the first time in non-classical PUF family proteins containing 11 Pumilio repeats arranged in an L shape. Using zoospore-specific dsRNA delivery technology to overcome the low uptake of dsRNA by oomycetes, we successfully developed an RNAi technique targeting Puf4 that effectively inhibited the pathogenicity of *Pythium* and *Phytophthora*. The application of RNAi technology to oomycete disease prevention and control was realized, and *Puf4* was identified as a potential target for such strategies.

PUFs, a class of highly conserved RNA-binding proteins, play essential roles in many processes in higher eukaryotes. In oomycetes, PUF1 protein adopts a crescent-shaped structure containing eight Pumilio repeat domains, mediating RNA binding on its concave surface. This structural feature plays a role in sexual reproduction by regulating the stability of key target mRNAs. For example, the *Puf1* members *PuM90*, *PsM90*, and *PlM90* are involved in sexual reproduction but not vegetative growth and virulence in *P*. *ultimum*, *Ph*. *sojae*, and *Peronophythora litchi* [5,35,36]. Similar to Puf-A and ScPuf6, the structure of Puf4 in oomycetes reveals a novel protein folding structure characterized by 11 Pumilio repeats arranged in an L shape, despite the prediction of only six, six, and seven Pumilio repeats in *Pythium ultimum*, *P*. *aphanidermatum*, and *Phytophthora sojae*, respectively, based on amino acid sequences. This finding suggests a highly conserved L-shaped architecture of Puf4 in oomycetes. Further experiments showed that knockout of *PuPuf4* or *PsPuf4* significantly attenuated pathogen virulence. Additionally, dsRNA targeting *PaPuf4* in *P*. *aphanidermatum* specifically suppressed its pathogenicity. These findings confirm the central role of Puf4 in the pathogenesis of oomycetes and highlight its L-shaped structure as important for its function as a non-canonical PUF protein. This unique structure may be linked to its ability to bind RNA or regulate other processes that are specific to oomycetes.

In contrast to the cytoplasmic localization of classical PUF proteins, PuPuf4, Puf-A, and ScPuf6 are localized in the nucleolus [15], and their mutants show distinct defects in rRNA processing. The processing of 45S pre-rRNA, an essential step for maturation of the ribosome, begins with independent cleavages at several sites driven by distinct endonucleases [37]. Transcriptional spacers (ITS1 and ITS2) and non-transcriptional spacers (3′ETS and 5′ETS) are degraded as non-functional maturation byproducts [31]. *ScPuf6* mutants accumulate 60S pre-ribosomes in the nucleus and show a slow growth phenotype, similar to ribosome biogenesis-defective mutants with 35S, 27S, and 7S pre-rRNA processing defects [15,30,38]. APUM24 binds ITS2 directly and we observed that processing intermediates, namely 27SB and 7S rRNAs, accumulated at higher levels in the *apum24-2* mutant than in the WT. Thus, APUM24 is likely involved in ITS2 removal [17]. RNA fragments originating from the 5′ETS, ITS1, and ITS2 were speciﬁcally enriched after coimmunoprecipitation with PuPuf4 in RIP assays (Fig 3B). In addition, *PuPuf4* deletion results in excessive accumulation of processing intermediates containing 5′ETS, ITS1, and ITS2 but not 3′ETS, indicating that PuPuf4 plays a role in the degradation of rRNA maturation byproducts and preserves the function of non-classical PUF proteins in pre-rRNA processing. This finding reveals that the L-shaped PUF protein has a highly conserved role in the regulation of rRNA processing. However, the rRNA cleavage sites in oomycetes are unknown, complicating the determination of which specific step of pre-RNA processing is abnormal and causes the accumulation of non-functional maturation byproducts. The accumulation of abnormal pre-rRNA nucleolus can activate the degradation of RNA exosomes, leading to the obstruction of 28S rRNA maturation, disrupting ribosome homeostasis and protein synthesis, and ultimately causing early developmental defects or lethal phenotypes in zebrafish and mice [39]. We speculate that the abnormal accumulation of precursor rRNA may disrupt ribosomal homeostasis by interfering with the assembly process of ribosomal proteins, but this mechanism still needs to be verified through subsequent experiments.

Nog2p, a highly conserved nuclear protein, contains a putative GTP‐binding site that is essential to maintaining normal rRNA levels*.* Deletion of *Nog2p* resulted in accumulation of the pre-rRNA maturation intermediates 27SB_S_ and 7S_S_. Co-IP and MS results indicated interaction between PuPuf4 and PuNog2, but the yeast two-hybrid results showed no interaction, indicating that PuPuf4 and PuNog2 cannot interact directly and another factor may act as a link between them. As biogenesis requires approximately 70 ribosome biogenesis factors that bind and release pre-60S at speciﬁc steps of the assembly pathway [40], proteins that directly interact with PuPuf4 require further exploration. We developed CRISPR/Cas9 system-mediated gene knockout and in situ complementation methods for *Pythium* [5]. At present, few relevant proteins, including PuPuf1 (PuM90) and PuLLP, have well understood functions [5,41]. In addition, genes that are suitable as RNAi targets are lacking, which has restricted the development of RNAi strategies against *Pythium*. More regulatory factors that influence pathogenicity and growth remain to be discovered. In this study, we identified PuPuf4 as a regulatory factor that impacts pathogenicity, thereby enriching the repertoire of RNA interference (RNAi) targets in *Pythium*. Despite repeated attempts at knockout of *PuNog2*, we were unable to generate a viable mutant, suggesting that the absence of *PuNog2* may be lethal to *P*. *ultimum*. Consequently, *PuNog2* has emerged as a promising new RNAi target gene that provides novel avenues for research and potential applications.

Generally, classical PUF proteins have eight Pumilio repeats organized into a crescent-shaped structure that bind to the core nucleotide sequence of 5′-UGUANAUA-3′ in mRNA 3′-UTRs. However, how these novel Pumilio repeat proteins interact with target RNA remains unclear, as prediction results show only five Pumilio repeats in PuPuf4 and APUM24 and six Pumilio repeats in ScPuf6, and these proteins bind RNA or DNA regardless of sequence [30]. In the present study, we identified an AG-rich motif in RIP-seq data and confirmed that PuPuf4 directly binds to this motif via EMSA and MST. Notably, L-shaped PUF proteins (including ScPuf6 and APUM24) also bind this motif, indicating that AG-rich motifs represent a novel binding signature for L-shaped PUF proteins. This discovery reveals that molecular recognition is conserved within the L-shaped PUF protein family. The identification of AG-enriched motifs provides new insights into the target selection mechanisms of these proteins, which may be linked to their distinct roles in cellular regulation. Interactions between L-shaped PUF proteins and AG-rich motifs through specific spatial structures suggest that their binding pockets may have unique electrostatic characteristics or spatial configurations that require further investigation, for example through cryo-electron microscopy.

RNAi-mediated gene silencing is an environmentally friendly method of disease control that has been applied to the prevention and control of various diseases; however, RNAi applications in oomycetes are lacking. Oomycetes have low dsRNA uptake efficiency due to their cell walls, and a few studies have reported the use of nanomaterials to enhance dsRNA uptake [27,28]. Using zoospores to overcome the delivery barrier imposed by oomycete cell walls, we significantly enhanced dsRNA delivery efficiency and successfully developed a nanomaterial-free dsRNA delivery technology. The findings indicate that dsRNA exerts its inhibitory effect on the translation process, as opposed to transcription, in *Phytophthora*. To the best of our knowledge, this is the first report of the use of dsRNA to control a *Pythium* pathogen. Genetic manipulation methods, such as gene knockout and silencing, have not yet been reported for *Pythium aphanidermatum*. The introduction of dsRNA to silence target genes, coupled with phenotypic analysis, elucidates the biological functions of genes and validates RNAi-based strategies as effective tools for controlling *Phytophthora* and *Pythium* pathogens.

**Fig 7:**
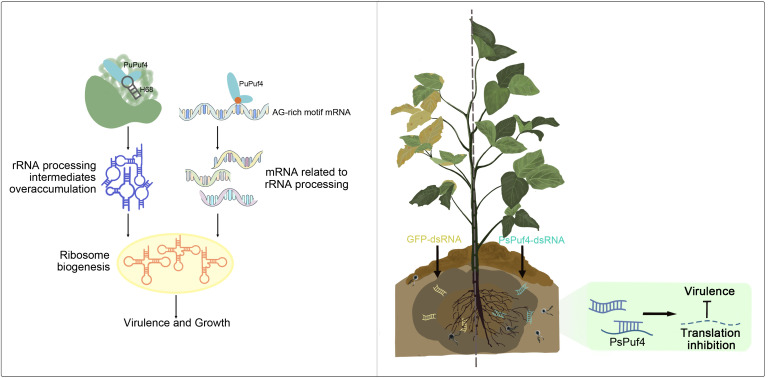
A proposed model for Puf4 function. PuPuf4 regulates the ribosome biogenesis by binding to the H68 component of 25S rRNA and AG-rich mRNA, influencing the pathogenicity and growth of the *P. ultimum*. Utilizing RNA interference technology, double-stranded RNA targeting *PsPuf4* inhibited the translation of the PsPuf4, significantly reducing the pathogenicity of *Ph. sojae*.

In summary, we propose a new model for the function of the non-classical PUF protein PuPuf4, a conserved rRNA processing regulator of pathogenicity and growth in oomycetes, thereby clarifying the regulation of rRNA processing in oomycetes (Fig 7). A novel binding motif for atypical PUF proteins was identified, providing clues into the interaction of non-classical PUF proteins with RNA. An RNAi strategy targeting *Puf4* was established for the control of *Phytophthora* and *Pythium* pathogens. The discovery of a unique rRNA processing mechanism in oomycetes and the *Puf4*-targeting RNA strategy developed in the current study may be useful for green management of devastating plant diseases.

## Materials and methods

### Source and culturing of strains

The strain of *Pythium ultimum* var. *ultimum* used as the WT strain was isolated from field soil in Shandong Province, China [42]. The genome-sequenced *Ph*. *sojae* strain P6497 (Race 2), provided by Dr. Brett Tyler (Department of Botany and Plant Pathology, Oregon State University, Corvallis, OR, USA), served as the WT strain of *Ph*. *sojae*. All strains employed in this study were routinely grown on 10% V8 agar medium at 25°C in the dark.

### CRISPR/Cas9-mediated gene knockout and complementation

Gene-deletion mutants were generated using the CRISPR-mediated gene replacement strategy. The *hph* gene, ligated with two 1.0-kb fragments flanking the target gene, was used as donor DNA for homology-directed repair (HDR). The primer combinations shown in S4A Fig were used to screen putative transformants. The F1/R1 primer set was used to screen for deletion of *PuPuf4* from the genomes of resistant transformants. Primer sets F2/R2 and F3/R3 were used to detect homologous recombination events (S5 Table). HDR events were analyzed through gDNA-PCR and Sanger sequencing to confirm that *PuPuf4* was cleanly replaced (S4A and S4C Fig). *PsPuf4* deletion mutants were constructed using the same strategy (S13 Fig). Polyethylene glycol-mediated protoplast transformation was conducted to introduce DNA into *P*. *ultimum* [5] *and Ph*. *sojae* [43]. For *PuPuf4* complementation, the knockout mutant was transformed using *NPTII* as the selection marker. The entire gene-coding region with mutated sgRNA sites, which was inserted between two 1.0-kb fragments flanking the target gene, was used as the donor DNA. The primer set F2/R2 was used to screen for the deletion of *hph* from the genomes of resistant transformants. The primer sets F1/R1 and F4/R4 (S4B and S4D Fig, S5 Table) were used to detect homologous recombination events.

### Mycelial growth and stress treatment

For growth rate analysis, the strains were cultured on V8 medium at 25°C in the dark. Sensitivity to stress was evaluated on V8 agar medium supplemented with various concentrations of stressors. To assess the growth rate at different temperatures, all tested strains were cultured on V8 medium at 15°C, 20°C, and 25°C in the dark. Colonial morphologies were photographed, and average colony diameter was determined from two orthogonal measurements. Relative mycelial growth was visualized as stress-treated colony diameters and corresponding non-treated colony diameters (Mock). These experiments were repeated three times. The results were compared using *t*-test in Excel software.

### Analysis of oospore development

To monitor and quantify oospore production, strains were grown on 10% V8 agar medium at 25°C in the dark. After 2, 5, and 14 days, the cultures were examined via microscopy (Olympus). Three random fields at 40 × magnification from each plate were selected for the counting of oospores. To allow for explicit observation of the oospores, five 5 × 5-mm hyphal plugs were cultivated in 8 mL of V8 broth in 90-mm Petri dishes for 7 days at 25°C in the dark. Oospores stained with lactophenol–trypan blue (10 mL lactic acid, 10 mL glycerol, 10 g phenol, and 10 mg trypan blue dissolved in 10 mL distilled water) were randomly selected for examination under an inverted microscope (Zeiss) [44]. Means and standard deviations were calculated using data from three biological replicates. Results were compared using *t*-test in Excel.

### Virulence assay

A virulence assay was performed using the Williams cultivar, as this cultivar is compatible with *P*. *ultimum and Ph*. *sojae*. Soybeans grown in a greenhouse at 25°C under a 16-h/8-h light/dark cycle for 4 days and 14 days were used for hypocotyl and leaf infection. Then, hyphal plugs (5 mm in diameter) were inoculated onto soybean hypocotyls for 24 h or leaves for 48 h prior to photography and sampling. Each strain was tested using at least five plants. Zoospores were retrieved as described previously and diluted to a concentration of 100 zoospores/10 µL. Etiolated seedlings were inoculated by pipetting 10 µL zoospore suspension onto the hypocotyl and incubating it at 25°C for 2 days, followed by photography. Virulence was quantified through determination of the ratio of *P*. *ultimum* or *Ph*. *sojae* DNA to soybean DNA in the infected plants, as measured through qRT-PCR. All assays were repeated independently at least three times. Results were compared via *t*-test in Excel.

### Co-immunoprecipitation assay

Constructs carrying GFP-tagged PuPuf4 and GFP-tag (control) in the pTOR vector were transformed into *P*. *ultimum*. Stable *P*. *ultimum* transformants were selected for total protein extraction and their total protein lysates were analyzed through western blotting using anti-GFP antibodies (Abmart Inc., Shanghai, China). This analysis revealed a band of 91 kDa, representing the PuPuf4-GFP fusion protein that was present in the transformants (S8B Fig). The total protein of the transformants was extracted from mycelium using protein lysis buffer [1 M Tris-Cl (pH 7.4), 1 M NaCl, 0.5 M ethylenediaminetetraacetic acid (EDTA), 1% Triton X-100] and mixed with anti-GFP agarose beads (GFP-Trap, Chromotek, Martinsried, Germany). The bead-bound proteins were then eluted and analyzed through MS.

For co-IP assays, FLAG-tagged PuNog2 and GFP-tagged PuPuf4 were stably co-expressed in *P*. *ultimum* (S9 Fig). Total protein was extracted from transformants expressing FLAG-tagged PuNog2 and GFP-tagged PuPuf4 and then incubated with GFP-Trap Agarose (ChromoTek, Martinsried, Germany) at 4°C for 6 h with rotation. The beads were collected through centrifugation at 2500 × g and washed three times in 1 mL of washing buffer (per manufacturer’s recommendations). Bound proteins were boiled for 5 min and detected via western blotting using anti-FLAG antibody (#A8592; Sigma-Aldrich) and anti-GFP antibodies (Abmart Inc., Shanghai, China).

### Electrophoretic mobility shift assay

EMSA was performed as described previously [5,45]. FAM-labeled probes comprised of the target sequences were synthesized by Genewiz (Suzhou, China). Labeled RNA fragments (50 ng) were mixed and incubated with various concentrations of purified PuPuf4 protein at 25°C for 30 min in EMSA/Gel-Shift Binding Buffer (Beyotime). The mixtures were then loaded onto a 1% agarose gel and electrophoresed for 1 h. EMSA signals (labeled RNA fragments) were detected using Alexa 488 with the VersaDoc imaging system (Bio-Rad, Philadelphia, PA, USA).

### Microscale thermophoresis

Binding of PuPuf4 protein to RNA probes labeled with FAM was detected through an MST assay using the Monolith NT.115 instrument (NanoTemper Technologies) as previously described [46]. A constant concentration (10 μM) of labeled RNA in MST buffer (50 mM Tris, pH 7.5, 150 mM NaCl, 10 mM MgCl_2_, and 0.05% Tween 20) was titrated against increasing concentrations of PuPuf4 protein dissolved in double-distilled water. MST premium-coated capillaries (Monolith NT.115 MO-K005) were used to load the samples into the MST instrument at 25°C using high MST power and 60% light-emitting diode power. Laser on and off times were set to 30 s and 5 s, respectively. All experiments were conducted in triplicate. Data were analyzed using NanoTemper Analysis software v. 1.2.101 (NanoTemper Technologies).

### RIP-seq and RIP-qPCR analysis

RIP-seq was performed as described previously with minor modifications [47–49]. Briefly, RIP was performed using *P*. *ultimum* transformants expressing GFP-tagged PuPuf4 (PuPuf4-GFP) and GFP-tag (control). Their mycelium was ground to powder using liquid nitrogen and suspended in 4 mL lysis buffer (50 mM Tris-HCl [pH 7.5], 150 mM KCl, 2 mM EDTA, 0.5% NP-40, 0.5 mM dithiothreitol, 1:100 v/v protease inhibitor cocktail [Sangon Biotech, Shanghai, China], 200 units/ml RNaseOUT [Invitrogen]), followed by simple sonication (30 s on, 30 s off, repeated twice). Homogenates were centrifuged for 20 min at 16000 × g and 4°C to clear the lysate. The supernatant was incubated with GFP-trap magnetic agarose (ChromoTek Ytmak-20) at 4°C overnight with rotation. RNA was extracted into TRIzol reagent after the bead-protein–RNA complexes were washed five times. RNA libraries were constructed and sequenced by BGI Genomics Co. (Shenzhen, China) or used for quantitative PCR analysis.

For RIP-qPCR, RNA was reverse transcribed into cDNA and subjected to qRT-PCR, followed by detection using SYBR green I fluorescent dye. Primer sequences are listed in S5 Table. Every RNA fragment analyzed was quantified based on three independent RIP analyses.

### Protein expression and purification

The coding sequences of PuPuf4 and PuPuf4 with three mutated amino acid residues were separately inserted into the pGEX-4T-2 vector containing the GST tag (GE Healthcare Life Science) for *in vitro* assays. The resulting plasmids (GST empty vector, GST-PuPuf4, and GST-PuPuf4^R172A Y208A R431A^) were transformed into *Escherichia coli* strain BL21 (DE3). A 500-mL culture of *E. coli* BL21(DE3) cells was grown at 37°C until the OD_600_ (optical density at 600 nm) reached 0.6, followed by induction of expression with 0.5 mM isopropyl β-d-1-thiogalactopyranoside (Sigma) for 4 h at 28°C. After lysis of cells, the recombinant proteins were purified on glutathione Sepharose beads (Sangon Biotech, Shanghai, China) and eluted with 5 mM glutathione dissolved in Tris-buffered saline. The concentration of purified proteins was determined using a bicinchoninic acid protein assay kit (Sangon Biotech). Protein purity was assessed via sodium dodecyl sulfate polyacrylamide gel electrophoresis.

### Yeast two-hybrid assay

Full-length cDNA of *PuPuf4* and *PuNog2* was cloned and inserted into the pGBKT7 (BD) and pGADT7 (AD) vectors, respectively. To examine protein interactions, the AD and BD constructs were co-transformed into the *Saccharomyces cerevisiae* strain Y2HGold and the transformants were grown on SD-Trp-Leu medium. Then, Trp+ and Leu+ transformants were isolated and plated on SD-Trp-Leu-His-Ade medium at 30°C for 4 days. Three independent biological replicates were assessed to confirm the results of each yeast two-hybrid assay.

### Figs and structural modeling

Figs of molecular structures were created using ChimeraX v1.6.1 (https://www.cgl.ucsf.edu/chimerax/). Sequence alignments were generated using CLUSTALW (https://www.ebi.ac.uk/jdispatcher/). Structural models of proteins were generated with Alphafold3 (https://alphafold3.org/) and visualized using ChimeraX v1.6.1.

### RNA-seq sampling and sequencing

RNA-seq samples of the WT and *PuPuf4* knockout mutant were collected from mycelium cultured on 10% V8 liquid medium at 25°C for 48 h. The hyphal plugs of *P. ultimum* were inoculated into soybean plants (Hefeng 47 variety). Samples were collected at 3, 6, 12, 24 and 36 hours after inoculation respectively for transcriptome sequencing analysis. Three independent biological replicates were analyzed for each treatment. RNA was extracted using the EZNA Total RNA Kit I (Omega BioTek, Norcross, GA, USA). RNA-seq was conducted by BGI Genomics Co. (Shenzhen, China) using the MGISEQ-2000 system with 100-bp paired-end reads. The filtered clean reads from RNA-seq have been deposited at the National Center for Biotechnology Information [BioProject ID: PRJNA1206542 (Temporary Submission ID: SUB14989373)].

The clean reads were aligned to the genome of *P*. *ultimum* var. *ultimum* (strain DAOM BR144) with TopHat v2.1.1 (https://ccb.jhu.edu). A total of two mismatches and gaps per read were allowed, and data were included in further analyses only if both reads in the pair were successfully mapped. Transcript abundance was indicated as fragments per kilobase of exon model per million mapped reads (FPKM). To identify DEGs, read counts for each gene model were obtained using featureCounts software (https://bioinf.wehi.edu.au); log_2_ fold change (log_2_FC) values and adjusted P-values were calculated using DESeq2 software (www.bioconductor.org); genes with adjusted P-value < 0.05 and absolute log_2_FC ≥ 2 were considered differentially expressed. To assess variability among samples, we performed hierarchical clustering and principal component analysis (using MEV 4.7.4; www.tm4.org) based on the FPKM values of genes.

### dsRNA synthesis and application

*In vitro* synthesis of dsRNA was conducted based on established protocols [24,50]. *In vitro* synthesis of dsRNA or Cy3-labeled dsRNA employed the MEGAscript RNAi Kit (Life Technologies, Carlsbad, CA, USA), and a dsRNA product labeled with Cy3 was generated using the control template provided in the kit. Primers used for *in vitro* synthesis of dsRNAs and the sequences of dsRNAs are listed in S5 Table. For confocal microscopic examination of fluorescein-labeled dsRNA uptake by mycelium and zoospores, 5 μL of 150 ng/μL labeled dsRNA was applied to mycelium and zoospores and incubated for 10–12 h, followed by confocal microscopic imaging. Zoospores and mycelium were treated with 75 U micrococcal nuclease enzyme at 37°C for 30 min to degrade dsRNA present on the surface of the zoospores or mycelium prior to observation. The fluorescent signals were analyzed using a Leica SP5 confocal microscope. For the pathogenicity assay, 10 μL of a zoospore suspension at 10^4^ per mL treated with *Puf4*-dsRNAs or *GFP*-specific dsRNA (control) for 30 min was inoculated onto soybean hypocotyls for 48 h or cucumber samples for 18 h or 36 h, and then lesion sizes were measured.

### Data access

The data that support the findings of this study are publicly available from NCBI BioProject database (https://www.ncbi.nlm.nih.gov/bioproject/) with the identifier PRJNA1206542.

## Supporting information

S1 Fig PuPuf4 protein forms a new PUM repeat fold. (A) Ribbon diagram of a protein structure of PuPuf4. Pumilio repeats are colored alternately green and orange in the N-terminal domain (N-R1-N-R3) and blue and pink in the C-terminal domain (C-R1-C-R8). N- and C-terminal pseudorepeats are indicated (N-R1′ and C-R8′, respectively). (B) Superposition of PuPuf4 repeats. Superposition of the Cα traces of Pumilio repeats from PuPuf4. Repeat C-R5 (pink) contains a 40-aa insertion between the α2 and α3 helices, Cα trace of PuPuf4 repeat C-R1. Repeat C-R1 is at the interface between the N- and C-terminal subdomains and does not align well with the other Pumilio repeats in PuPuf4.
(DOCX)

S2 Fig The protein structures of PuM90 and PuPuf4, along with associated RNA bases. (A-B) Protein structure of the Puf RNA binding domain in PuM90, individual amino acids predicted to interact with RNA are colored in red, the predicted RNA bases targeted by each Pumilio repeat are shown. (C-D) Alignment of α2 helix amino acid sequences of PuPuf4 (red) and PuM90 (blue). The five-residue sequences that recognize RNA in PuPuf4 are numbered 1–5 above the sequences. Residues in PuPuf4 that recognize the edges of bases (first and fifth positions) are highlighted green and blue, respectively, whereas residues that stack with RNA bases (second position) are highlighted magenta. Equivalent positions in PuM90 are indicated.
(DOCX)

S3 Fig Protein Structures of PsPuf4 and PaPuf4. (A) Domain structures of PsPuf4 and PaPuf4 predicted by SMART (http://smart.embl-heidelberg.de/) based on amino acid sequences. (B) Ribbon diagrams showing the structural features of PsPuf4 and PaPuf4.
(DOCX)

S4 Fig CRISPR/Cas9-mediated *PuPuf4* gene knockout and complementation. (A) Schematic diagram of homology-directed repair-mediated modification of the target gene, an ‘all-in-one’ plasmid (pYF515) harboring both Cas9 and sgRNA cassettes was co-transformed with a plasmid (pBS-SK II+) containing homologous donor DNA *hph* with *PuPuf4* flanking sequences. Locations of the primers used to screen the HRR mutants and Sanger sequencing traces of junction regions confirming that the *PuPuf4* ORF was precisely replaced. (B) Analysis of genomic DNA from the wildtype (WT), empty-vector control line (EV), and *PuPuf4*-knockout mutants (Δ*PuPuf4*-1/2/3) using the primers shown at the top and actin primers as a positive control. (C) Schematic representation of the Δ*PuPuf4* mutant complementation strategy and the plasmids used for second transformation in *Pythium*. *PuPuf4-m* with two black triangles indicates *PuPuf4* modified with two sgRNA targeting sequences. Locations of the primers used to screen the complementation mutants and Sanger sequencing traces of junction regions confirming that the *PuPuf4* ORF was precisely complemented. (D) Analysis of genomic DNA from the wild-type (WT), complemented transformants (Δ*PuPuf4*-C1/2), and empty control line of Δ*PuPuf4* (ΔPuPuf4-EV) using the primers shown at the top and actin primers as a positive control.
(DOCX)

S5 Fig Growth characteristics of WT, EV, ΔPuPuf4, ΔPuPuf4-EV and ΔPuPuf4-Complement on 10% V8 agar medium only and supplemented with sorbitol (0.5 M), NaCl (0.4 M) and H_2_O_2_ (6.5 mM). (DOCX)

S6 Fig Growth of the ΔPuPuf4 mutants showed a more severe reduction at low temperature compared with WT. (A-B) Growth characteristics a) and Colony size (B) of WT, EV, ΔPuPuf4, ΔPuPuf4–EV and ΔPuPuf4-Complement on 10% V8 agar medium in 25°C, 20°C and 15°C. Asterisks indicate significant differences comparing with WT at P < 0.01 (**).
(DOCX)

S7 Fig Oospore formation of WT, EV, ΔPuPuf4, ΔPuPuf4–EV and ΔPuPuf4-complement grown on 10% V8 solid medium for 2d, 5d and 14d (Lines one, two and three), Morphology of oospore from 7-day-old cultures grown in V8 liquid medium (The fourth line). Bar, 20 μm.
(DOCX)

S8 Fig The PuPuf4 protein fused with GFP was expressed in the wild type of *P. ultimum.* (A) Schematic representation of carrier expressing PuPuf4 C-terminal fusion GFP and only GFP in the wild type of *P. ultimum.* (B) The extracted transformant protein was subjected to denaturing gel electrophoresis, then hybridized with GFP antibody, and detected by western blot. The results showed that PuPuf4-GFP had been expressed in *P. ultimum.*
(DOCX)

S9 Fig Expression of PuNog2-FLAG and PuPuf4-GFP in *P. ultimum*, extracts of *P. ultimum* were resolved by SDS-PAGE on a 12.5% acrylamide gel, and the presence of FLAG-tagged proteins and GFP-tagged proteins were detected by western blot analysis using FLAG and GFP antibody. The size of relevant molecular weight markers (MWM) is indicated on the left.
(DOCX)

S10 Fig PuPuf4 and PuNog2 interaction verification experiments. (A) Validation of the association between PuPuf4 and PuNog2 *in vivo*. Co-immunoprecipitations (Co-IP) were performed in extracts of *P. ultimum* mycelium expressing PuNog2-FLAG with PuPuf4-GFP. The presence of FLAG-tagged proteins was detected by western blot analysis using a FLAG antibody. The bands detected with anti-GFP were quantified with the ODYSSEY infrared imaging system (application software version 2.1). (B) The yeast two-hybrid (Y2H) assay indicated that PuPuf4 can not interact with PuNog2. Ten-fold serial dilutions of yeast cells transferred with the bait and prey construct were assayed for growth on SD-Leu-Trp-His-Ade plates. A pair of plasmids, pGBKT7-53 and pGADT7-T was used as the positive control, while pGBKT7-Lam and pGADT7-T was used as the negative control.
(DOCX)

S11 Fig Electrostatic surface representation of a structure of ScPuf6. (DOCX)

S12 Fig Amino acid alignment of PuPuf4, ScPuf6 and APUM24. (DOCX)

S13 Fig CRISPR-mediated gene replacement of *PsPuf4.* (A) Schematic diagram of homology-directed repair-mediated modification of the target gene, an ‘all-in-one’ plasmid (pYF515) harboring both Cas9 and sgRNA cassettes was co-transformed with a plasmid (pBS-SK II+) containing homologous donor DNA *hph* with *PsPuf4* flanking sequences. Locations of the primers used to screen the HRR mutants and Sanger sequencing traces of junction regions confirming that the *PsPuf4* ORF was precisely replaced. (B) Analysis of genomic DNA from the wildtype (WT), empty-vector control line (EV), and *PsPuf4*-knockout mutants (Δ*PsPuf4-*1/2/3) using the primers shown at the top and actin primers as a positive control.
(DOCX)

S14 Fig Growth characteristics of WT, EV, ΔPsPuf4 on 10% V8 agar medium only and supplemented with sorbitol (1 M), NaCl (0.6 M) and H_2_O_2_ (5 mM). (DOCX)

S15 Fig Oospore formation of WT, EV and ΔPsPuf4 after culture for 2d, 6d and 14d. (A) Oospore formation of WT, EV, ΔPsPuf4 grown on 10% V8 solid medium for 2d, 6d and 14d (Lines one, two and three), Morphology of oospore from 7-day-old cultures grown in V8 liquid medium (The fourth line). Bar, 20 μm. (B) Oospore number cultured for 2d, 6d and 14d. Asterisks indicate significant differences comparing with WT at P < 0.01 (**).
(DOCX)

S16 Fig *PsPuf4* is required for vegetative growth and virulence of *Ph. sojae* and *PsPuf4*-dsRNA compromises the virulence of *Ph. sojae* on soybean. (A) Infection lesions on soybean hypocotyl 48 h post-inoculation. (B) Relative *Ph. sojae* biomass detected through qRT-PCR at 48 h after hypocotyl infection. Asterisks indicate significant differences comparing with WT at P < 0.01 (**).
(DOCX)

S17 Fig The transcript level of *PsPuf4* in both *PsPuf4*-dsRNA, *GFP*-dsRNA and H_2_O treated zoospore were measured. (DOCX)

S1 Table PUF proteins identified in oomycetes, fungi, *Arabidopsis thaliana*, and humans. (XLSX)

S2 Table PuPuf4 binding peaks identified by RIP-seq. (XLSX)

S3 Table PuPuf4-interacting proteins identified via mass spectrometry. (XLSX)

S4 Table Differentially regulated genes when *PuPuf4* was knocked out. (XLSX)

S5 Table Primers and sgRNAs used in this study. (XLSX)
