# Supplementary material for: A non-classical PUF family protein in oomycetes functions as a pre-rRNA processing regulator and a target for RNAi-based disease control
Source: PLoS Pathog. 2025 Jul 31;21(7):e1013379. doi: 10.1371/journal.ppat.1013379 (PMC12324679; doi:10.1371/journal.ppat.1013379)
Supplement: S1 Fig — (A) Ribbon diagram of a protein structure of PuPuf4. Pumilio repeats are colored alternately green and orange in the N-terminal domain (N-R1-N-R3) and blue and pink in the C-terminal domain (C-R1-C-R8). N- and C-terminal pseudorepeats are indicated (N-R1′ and C-R8′, respectively). (B) Superposition of PuPuf4 repeats. Superposition of the Cα traces of Pumilio repeats from PuPuf4. Repeat C-R5 (pink) contains a 40-aa insertion between the α2 and α3 helices, Cα trace of PuPuf4 repeat C-R1. Repeat C-R1 is at the interface between the N- and C-terminal subdomains and does not align well with the other Pumilio repeats in PuPuf4. (DOCX) [file ppat.1013379.s001.docx]

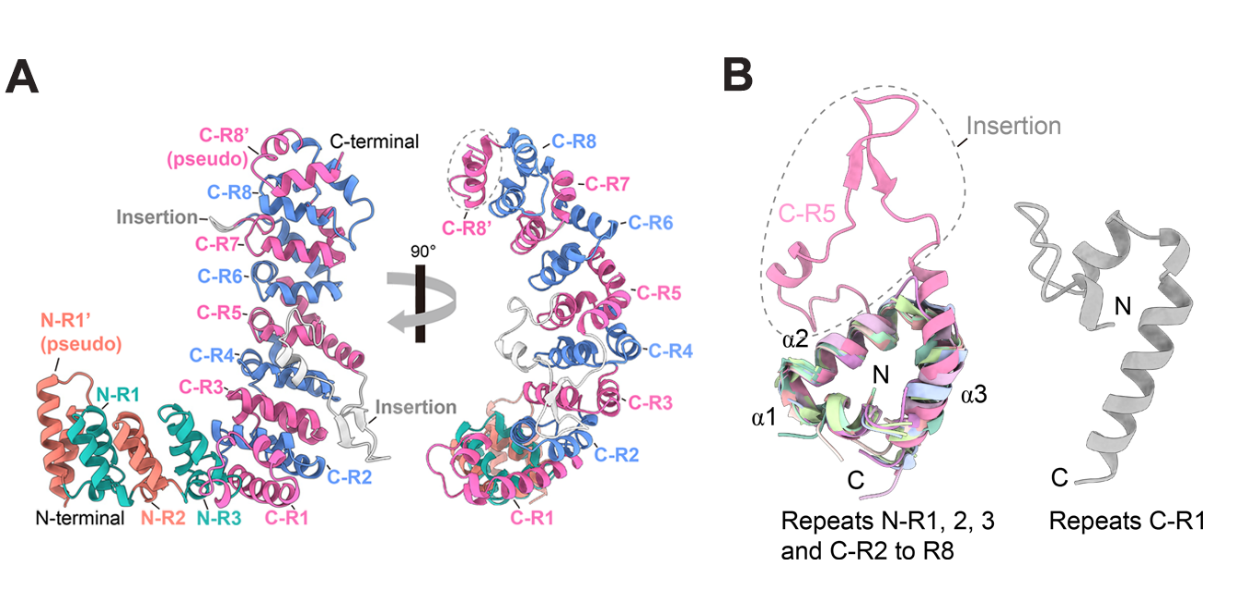


**S1 Fig.** **PuPuf4 protein forms a new PUM repeat fold.** (A) Ribbon diagram of a protein structure of PuPuf4. Pumilio repeats are colored alternately green and orange in the N-terminal domain (N-R1-N-R3) and blue and pink in the C-terminal domain (C-R1-C-R8). N- and C-terminal pseudorepeats are indicated (N-R1′ and C-R8′, respectively). (B) Superposition of PuPuf4 repeats. Superposition of the Cα traces of Pumilio repeats from PuPuf4. Repeat C-R5 (pink) contains a 40-aa insertion between the α2 and α3 helices, Cα trace of PuPuf4 repeat C-R1. Repeat C-R1 is at the interface between the N- and C-terminal subdomains and does not align well with the other Pumilio repeats in PuPuf4.
