# Supplementary material for: A non-classical PUF family protein in oomycetes functions as a pre-rRNA processing regulator and a target for RNAi-based disease control
Source: PLoS Pathog. 2025 Jul 31;21(7):e1013379. doi: 10.1371/journal.ppat.1013379 (PMC12324679; doi:10.1371/journal.ppat.1013379)
Supplement: S2 Fig — (A-B) Protein structure of the Puf RNA binding domain in PuM90, individual amino acids predicted to interact with RNA are colored in red, the predicted RNA bases targeted by each Pumilio repeat are shown. (C-D) Alignment of α2 helix amino acid sequences of PuPuf4 (red) and PuM90 (blue). The five-residue sequences that recognize RNA in PuPuf4 are numbered 1–5 above the sequences. Residues in PuPuf4 that recognize the edges of bases (first and fifth positions) are highlighted green and blue, respectively, whereas residues that stack with RNA bases (second position) are highlighted magenta. Equivalent positions in PuM90 are indicated. (DOCX) [file ppat.1013379.s002.docx]

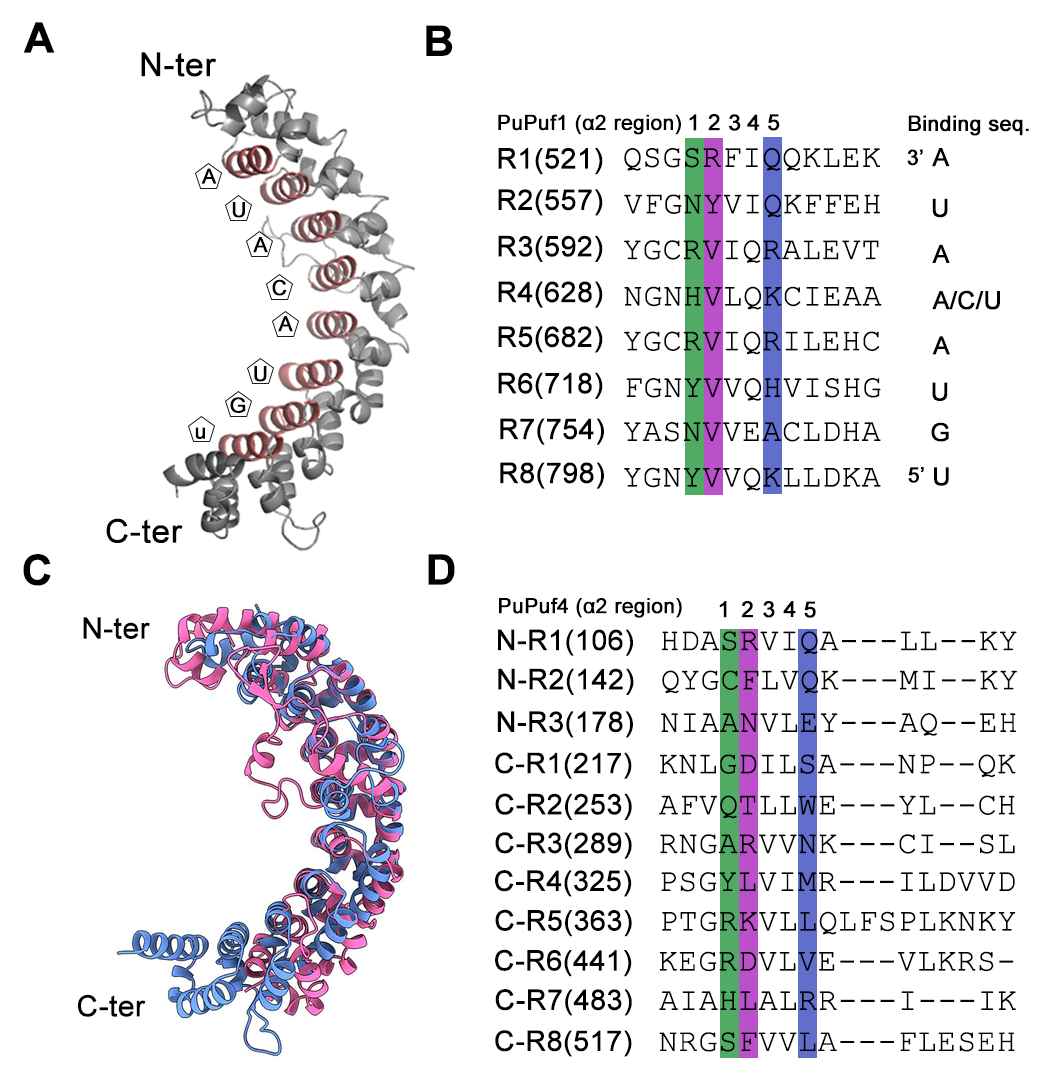


**S2 Fig. The protein structures of PuPuf1 and PuPuf4, along with associated RNA bases. (**A-B) Protein structure of the Puf RNA binding domain in PuM90, individual amino acids predicted to interact with RNA are colored in red, the predicted RNA bases targeted by each Pumilio repeat are shown. (C-D) Alignment of α2 helix amino acid sequences of PuPuf4 (red) and PuM90 (blue). The five-residue sequences that recognize RNA in PuPuf4 are numbered 1-5 above the sequences. Residues in PuPuf4 that recognize the edges of bases (first and fifth positions) are highlighted green and blue, respectively, whereas residues that stack with RNA bases (second position) are highlighted magenta. Equivalent positions in PuM90 are indicated.
