# Supplementary material for: A non-classical PUF family protein in oomycetes functions as a pre-rRNA processing regulator and a target for RNAi-based disease control
Source: PLoS Pathog. 2025 Jul 31;21(7):e1013379. doi: 10.1371/journal.ppat.1013379 (PMC12324679; doi:10.1371/journal.ppat.1013379)
Supplement: S3 Fig — (A) Domain structures of PsPuf4 and PaPuf4 predicted by SMART (http://smart.embl-heidelberg.de/) based on amino acid sequences. (B) Ribbon diagrams showing the structural features of PsPuf4 and PaPuf4. (DOCX) [file ppat.1013379.s003.docx]

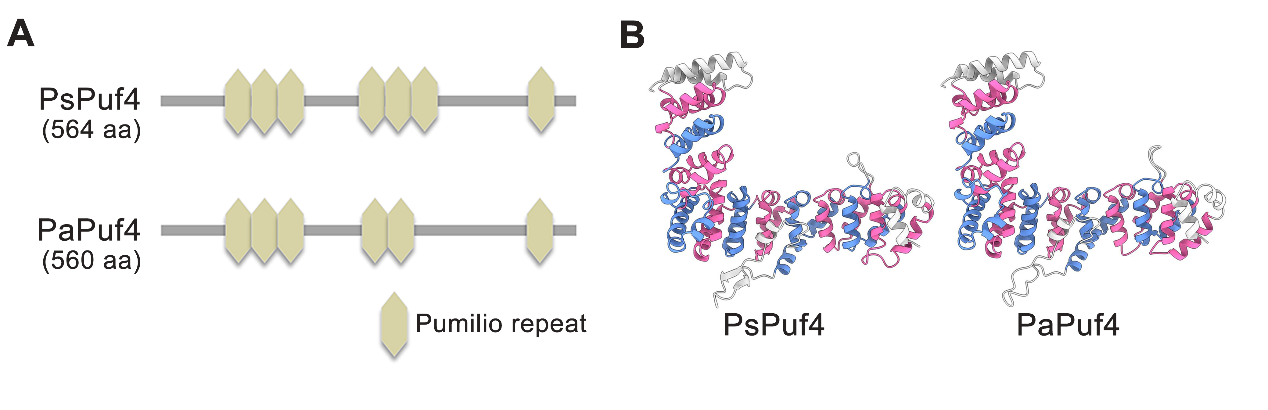


**S3 Fig. Protein Structures of PsPuf4 and PaPuf4.** (A) Domain structures of PsPuf4 and PaPuf4 predicted by SMART (<http://smart.embl-heidelberg.de/>) based on amino acid sequences. (B) Ribbon diagrams showing the structural features of PsPuf4 and PaPuf4.
