# Supplementary material for: A non-classical PUF family protein in oomycetes functions as a pre-rRNA processing regulator and a target for RNAi-based disease control
Source: PLoS Pathog. 2025 Jul 31;21(7):e1013379. doi: 10.1371/journal.ppat.1013379 (PMC12324679; doi:10.1371/journal.ppat.1013379)
Supplement: S4 Fig — (A) Schematic diagram of homology-directed repair-mediated modification of the target gene, an ‘all-in-one’ plasmid (pYF515) harboring both Cas9 and sgRNA cassettes was co-transformed with a plasmid (pBS-SK II+) containing homologous donor DNA hph with PuPuf4 flanking sequences. Locations of the primers used to screen the HRR mutants and Sanger sequencing traces of junction regions confirming that the PuPuf4 ORF was precisely replaced. (B) Analysis of genomic DNA from the wildtype (WT), empty-vector control line (EV), and PuPuf4-knockout mutants (ΔPuPuf4-1/2/3) using the primers shown at the top and actin primers as a positive control. (C) Schematic representation of the ΔPuPuf4 mutant complementation strategy and the plasmids used for second transformation in Pythium. PuPuf4-m with two black triangles indicates PuPuf4 modified with two sgRNA targeting sequences. Locations of the primers used to screen the complementation mutants and Sanger sequencing traces of junction regions confirming that the PuPuf4 ORF was precisely complemented. (D) Analysis of genomic DNA from the wild-type (WT), complemented transformants (ΔPuPuf4-C1/2), and empty control line of ΔPuPuf4 (ΔPuPuf4-EV) using the primers shown at the top and actin primers as a positive control. (DOCX) [file ppat.1013379.s004.docx]

**
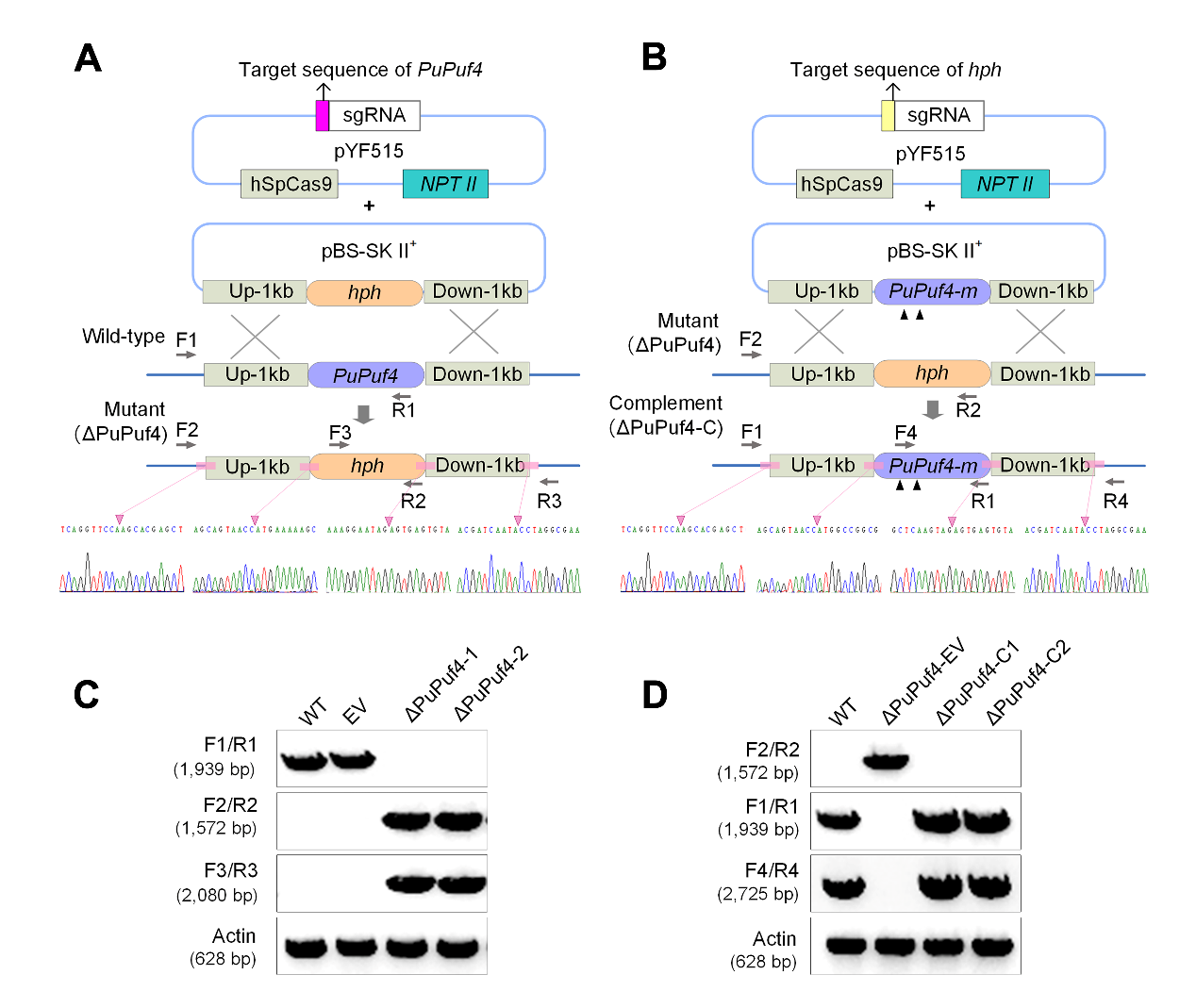
**

**S4 Fig. CRISPR/Cas9-mediated *PuPuf4* gene knockout and complementation.** (A) Schematic diagram of homology-directed repair-mediated modification of the target gene, an ‘all-in-one’ plasmid (pYF515) harboring both Cas9 and sgRNA cassettes was co-transformed with a plasmid (pBS-SK II+) containing homologous donor DNA *hph* with *PuPuf4* flanking sequences. Locations of the primers used to screen the HRR mutants and Sanger sequencing traces of junction regions confirming that the *PuPuf4* ORF was precisely replaced. (B) Analysis of genomic DNA from the wildtype (WT), empty-vector control line (EV), and *PuPuf4*-knockout mutants (Δ*PuPuf4*-1/2/3) using the primers shown at the top and actin primers as a positive control. (C) Schematic representation of the Δ*PuPuf4* mutant complementation strategy and the plasmids used for second transformation in *Pythium*. *PuPuf4-m* with two black triangles indicates *PuPuf4* modified with two sgRNA targeting sequences. Locations of the primers used to screen the complementation mutants and Sanger sequencing traces of junction regions confirming that the *PuPuf4* ORF was precisely complemented. (D) Analysis of genomic DNA from the wild-type (WT), complemented transformants (Δ*PuPuf4*-C1/2), and empty control line of Δ*PuPuf4* (ΔPuPuf4-EV) using the primers shown at the top and actin primers as a positive control.
