# Supplementary material for: A non-classical PUF family protein in oomycetes functions as a pre-rRNA processing regulator and a target for RNAi-based disease control
Source: PLoS Pathog. 2025 Jul 31;21(7):e1013379. doi: 10.1371/journal.ppat.1013379 (PMC12324679; doi:10.1371/journal.ppat.1013379)
Supplement: S5 Fig — (DOCX) [file ppat.1013379.s005.docx]

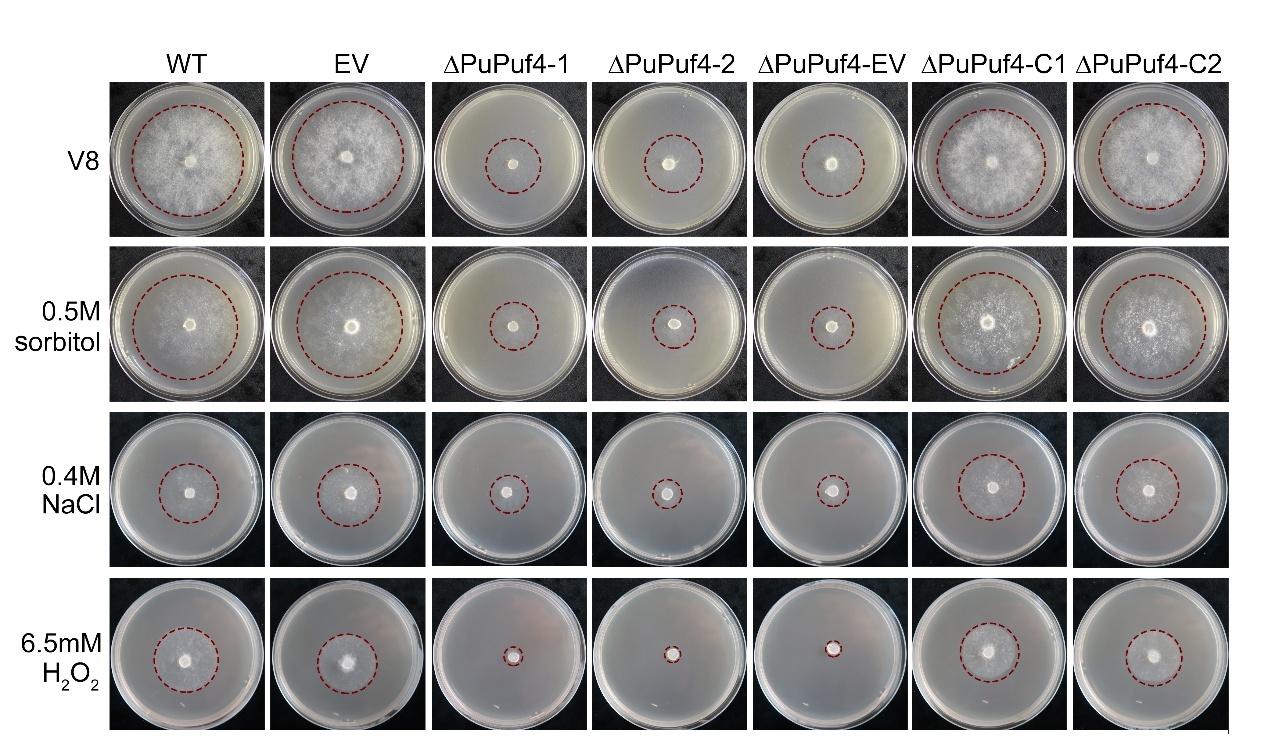


**S5 Fig.** Growth characteristics of WT, EV, ΔPuPuf4, ΔPuPuf4-EV and ΔPuPuf4-Complement on 10% V8 agar medium only and supplemented with sorbitol (0.5 M), NaCl (0.4 M) and H_2_O_2_ (6.5 mM).
