# Supplementary material for: A non-classical PUF family protein in oomycetes functions as a pre-rRNA processing regulator and a target for RNAi-based disease control
Source: PLoS Pathog. 2025 Jul 31;21(7):e1013379. doi: 10.1371/journal.ppat.1013379 (PMC12324679; doi:10.1371/journal.ppat.1013379)
Supplement: S6 Fig — (A-B) Growth characteristics a) and Colony size (B) of WT, EV, ΔPuPuf4, ΔPuPuf4–EV and ΔPuPuf4-Complement on 10% V8 agar medium in 25°C, 20°C and 15°C. Asterisks indicate significant differences comparing with WT at P < 0.01 (**). (DOCX) [file ppat.1013379.s006.docx]

**
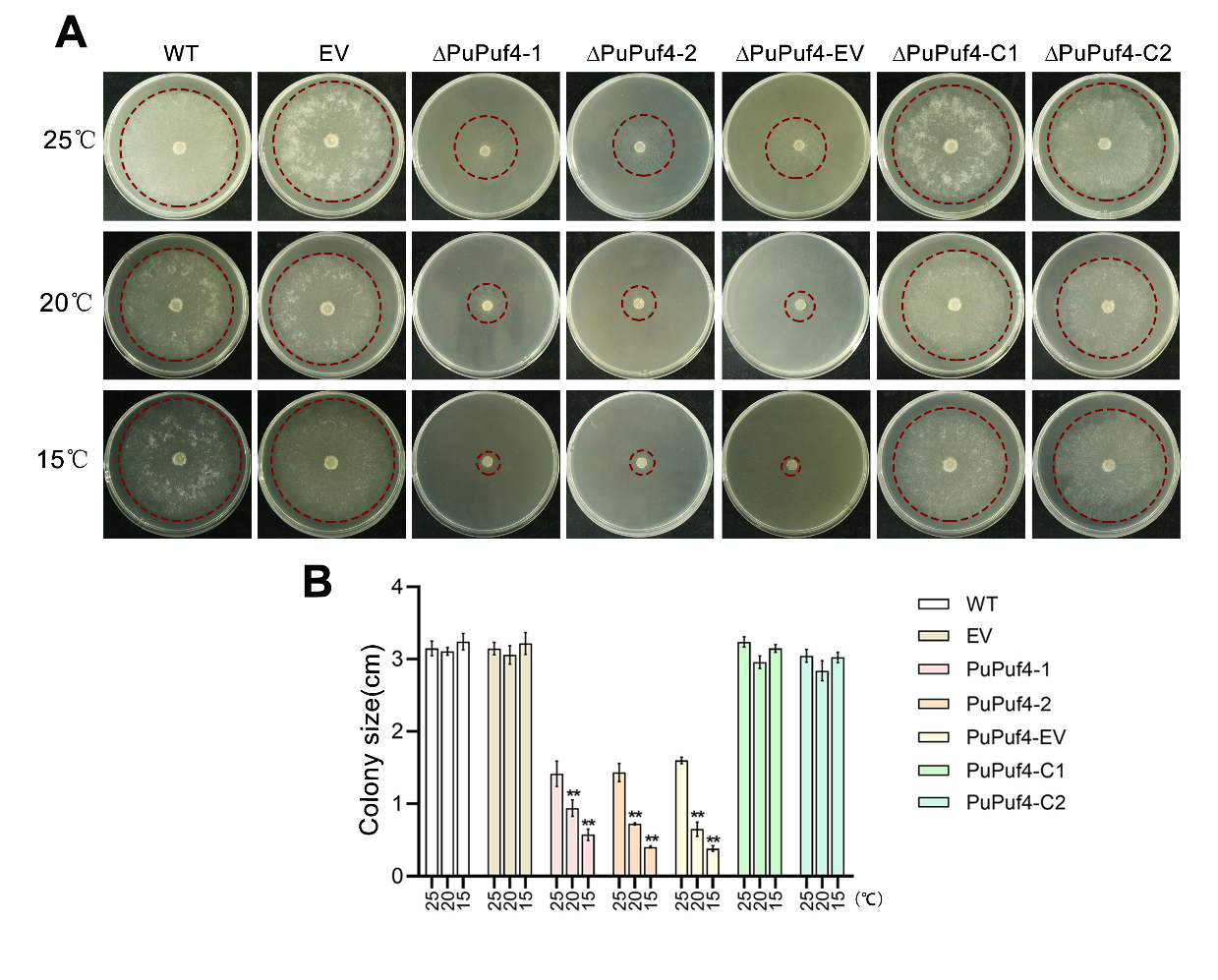
**

**S6 Fig. Growth of the ΔPuPuf4 mutants showed a more severe reduction at low temperature compared with WT**. (A-B) Growth characteristics a) and Colony size (B) of WT, EV, ΔPuPuf4, ΔPuPuf4–EV and ΔPuPuf4-Complement on 10% V8 agar medium in 25℃, 20℃ and 15℃. Asterisks indicate significant differences comparing with WT at P < 0.01 (**).
