# Supplementary material for: A non-classical PUF family protein in oomycetes functions as a pre-rRNA processing regulator and a target for RNAi-based disease control
Source: PLoS Pathog. 2025 Jul 31;21(7):e1013379. doi: 10.1371/journal.ppat.1013379 (PMC12324679; doi:10.1371/journal.ppat.1013379)
Supplement: S7 Fig — Bar, 20 μm. (DOCX) [file ppat.1013379.s007.docx]

**
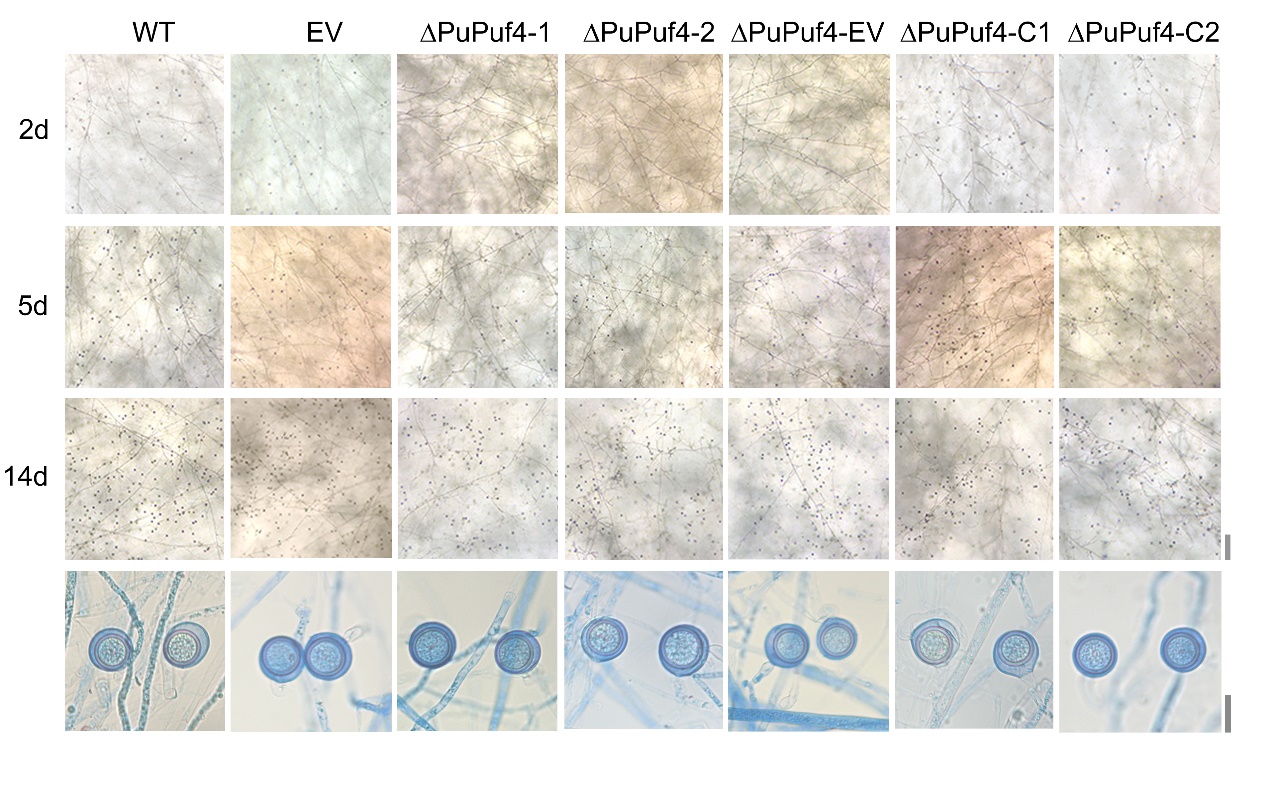
**

**S7 Fig.** Oospore formation of WT, EV, ΔPuPuf4, ΔPuPuf4–EV and ΔPuPuf4-complement grown on 10% V8 solid medium for 2d, 5d and 14d (Lines one, two and three), Morphology of oospore from 7-day-old cultures grown in V8 liquid medium (The fourth line). Bar, 20 μm.
