# Supplementary material for: A non-classical PUF family protein in oomycetes functions as a pre-rRNA processing regulator and a target for RNAi-based disease control
Source: PLoS Pathog. 2025 Jul 31;21(7):e1013379. doi: 10.1371/journal.ppat.1013379 (PMC12324679; doi:10.1371/journal.ppat.1013379)
Supplement: S8 Fig — (A) Schematic representation of carrier expressing PuPuf4 C-terminal fusion GFP and only GFP in the wild type of P. ultimum. (B) The extracted transformant protein was subjected to denaturing gel electrophoresis, then hybridized with GFP antibody, and detected by western blot. The results showed that PuPuf4-GFP had been expressed in P. ultimum. (DOCX) [file ppat.1013379.s008.docx]

**
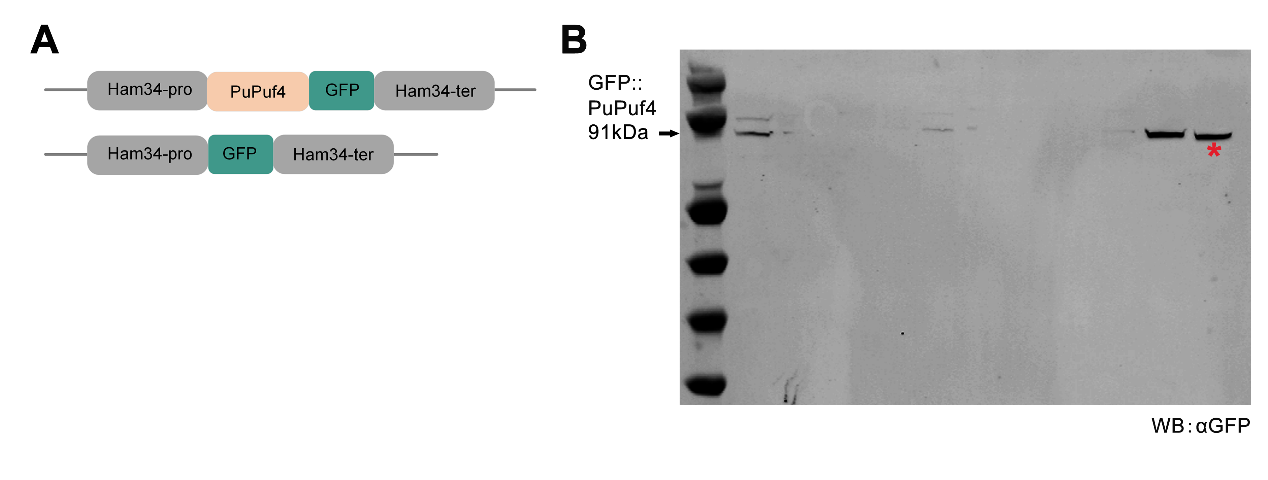
**

**S8 Fig. The PuPuf4 protein fused with GFP was expressed in the wild type of *P. ultimum*.** (A) Schematic representation of carrier expressing PuPuf4 C-terminal fusion GFP and only GFP in the wild type of *P. ultimum.* (B) The extracted transformant protein was subjected to denaturing gel electrophoresis, then hybridized with GFP antibody, and detected by western blot. The results showed that PuPuf4-GFP had been expressed in *P. ultimum.*
