# Supplementary material for: A non-classical PUF family protein in oomycetes functions as a pre-rRNA processing regulator and a target for RNAi-based disease control
Source: PLoS Pathog. 2025 Jul 31;21(7):e1013379. doi: 10.1371/journal.ppat.1013379 (PMC12324679; doi:10.1371/journal.ppat.1013379)
Supplement: S9 Fig — The size of relevant molecular weight markers (MWM) is indicated on the left. (DOCX) [file ppat.1013379.s009.docx]

**
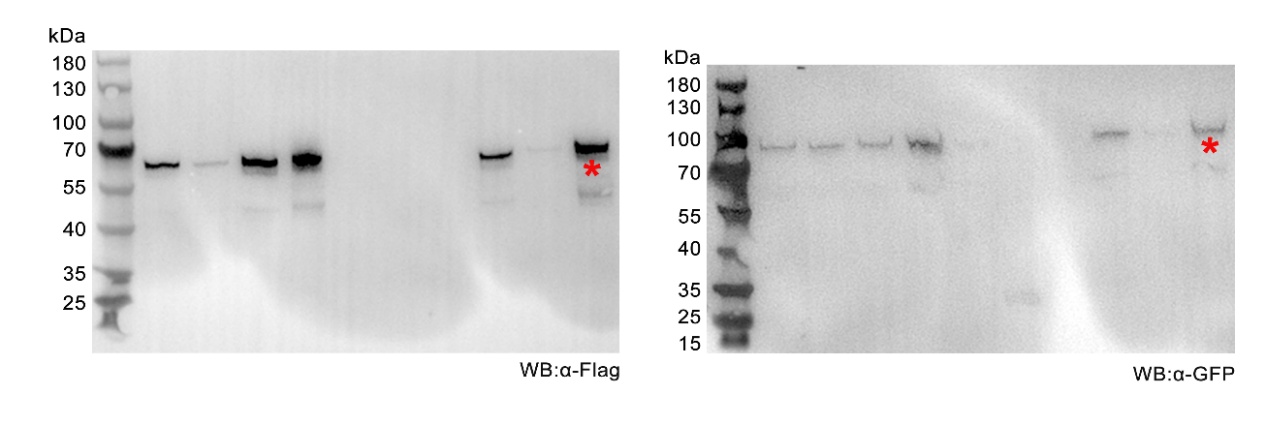
**

**S9 Fi****g.** Expression of PuNog2-FLAG and PuPuf4-GFP in *P. ultimum*, extracts of *P. ultimum* were resolved by SDS-PAGE on a 12.5% acrylamide gel, and the presence of FLAG-tagged proteins and GFP-tagged proteins were detected by western blot analysis using FLAG and GFP antibody. The size of relevant molecular weight markers (MWM) is indicated on the left.
