# Supplementary material for: A non-classical PUF family protein in oomycetes functions as a pre-rRNA processing regulator and a target for RNAi-based disease control
Source: PLoS Pathog. 2025 Jul 31;21(7):e1013379. doi: 10.1371/journal.ppat.1013379 (PMC12324679; doi:10.1371/journal.ppat.1013379)
Supplement: S10 Fig — (A) Validation of the association between PuPuf4 and PuNog2 in vivo. Co-immunoprecipitations (Co-IP) were performed in extracts of P. ultimum mycelium expressing PuNog2-FLAG with PuPuf4-GFP. The presence of FLAG-tagged proteins was detected by western blot analysis using a FLAG antibody. The bands detected with anti-GFP were quantified with the ODYSSEY infrared imaging system (application software version 2.1). (B) The yeast two-hybrid (Y2H) assay indicated that PuPuf4 can not interact with PuNog2. Ten-fold serial dilutions of yeast cells transferred with the bait and prey construct were assayed for growth on SD-Leu-Trp-His-Ade plates. A pair of plasmids, pGBKT7-53 and pGADT7-T was used as the positive control, while pGBKT7-Lam and pGADT7-T was used as the negative control. (DOCX) [file ppat.1013379.s010.docx]

**
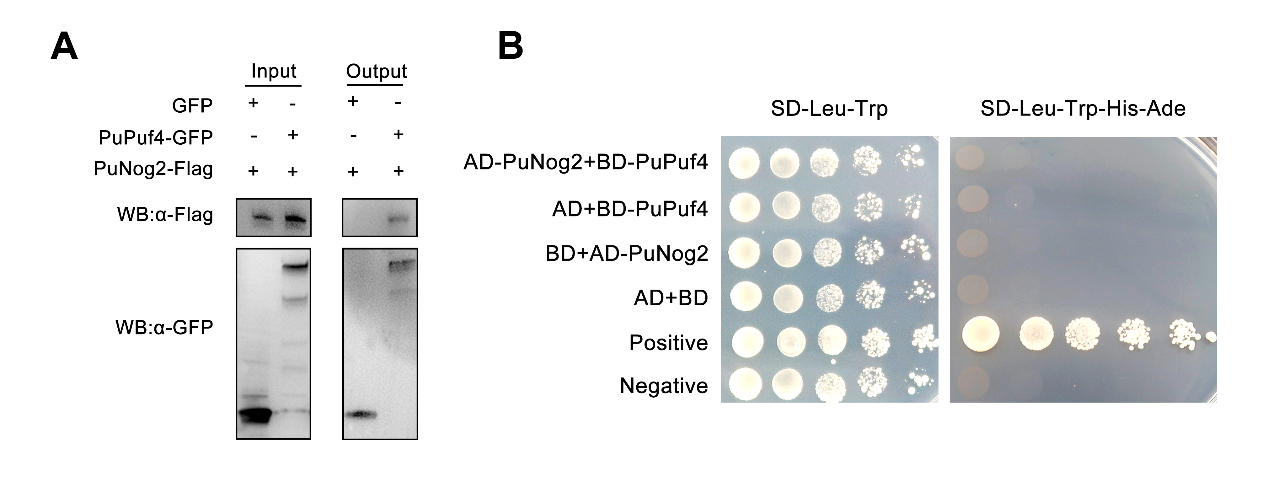
**

**S10 Fig. PuPuf4 and** **PuNog2 interaction verification experiments.** (A) Validation of the association between PuPuf4 and PuNog2 *in vivo*. Co-immunoprecipitations (Co-IP) were performed in extracts of *P. ultimum* mycelium expressing PuNog2-FLAG with PuPuf4-GFP. The presence of FLAG-tagged proteins was detected by western blot analysis using a FLAG antibody. The bands detected with anti-GFP were quantified with the ODYSSEY infrared imaging system (application software version 2.1). (B) The yeast two-hybrid (Y2H) assay indicated that PuPuf4 can not interact with PuNog2. Ten-fold serial dilutions of yeast cells transferred with the bait and prey construct were assayed for growth on SD-Leu-Trp-His-Ade plates. A pair of plasmids, pGBKT7-53 and pGADT7-T was used as the positive control, while pGBKT7-Lam and pGADT7-T was used as the negative control.
