# Supplementary figures and images for: A non-classical PUF family protein in oomycetes functions as a pre-rRNA processing regulator and a target for RNAi-based disease control
Source: PLoS Pathog. 2025 Jul 31;21(7):e1013379. doi: 10.1371/journal.ppat.1013379 (PMC12324679; doi:10.1371/journal.ppat.1013379)

**
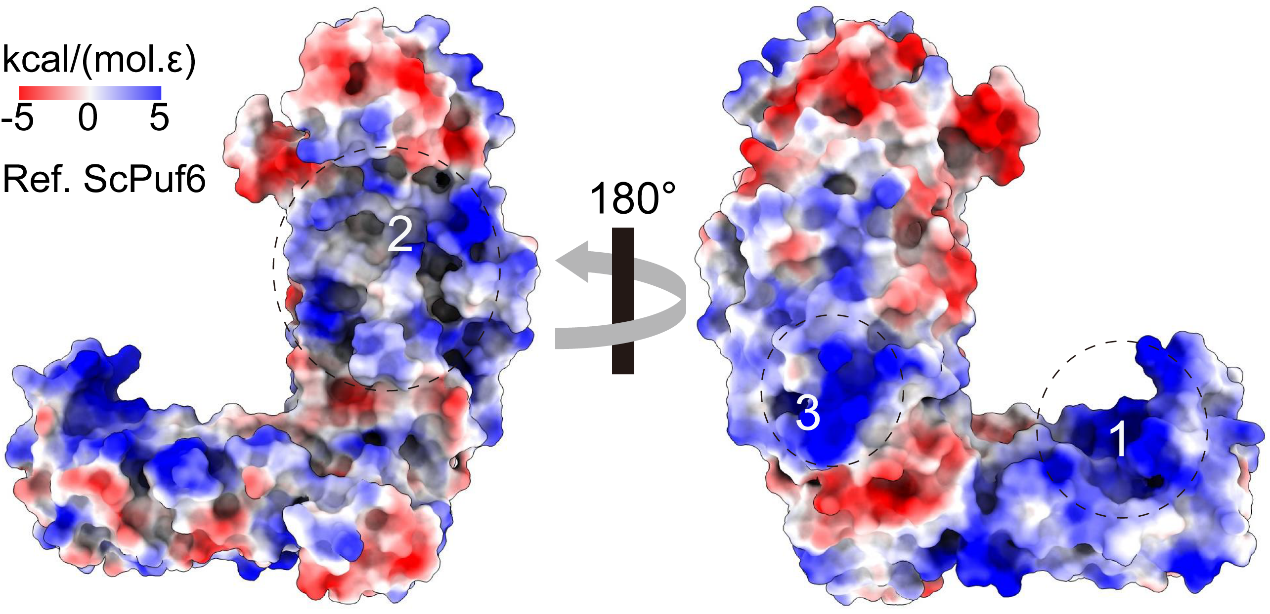
**

**S11 Fig. Electrostatic surface representation of a structure of ScPuf6.**

Supplement: S11 Fig — (DOCX) [file ppat.1013379.s011.docx]

**
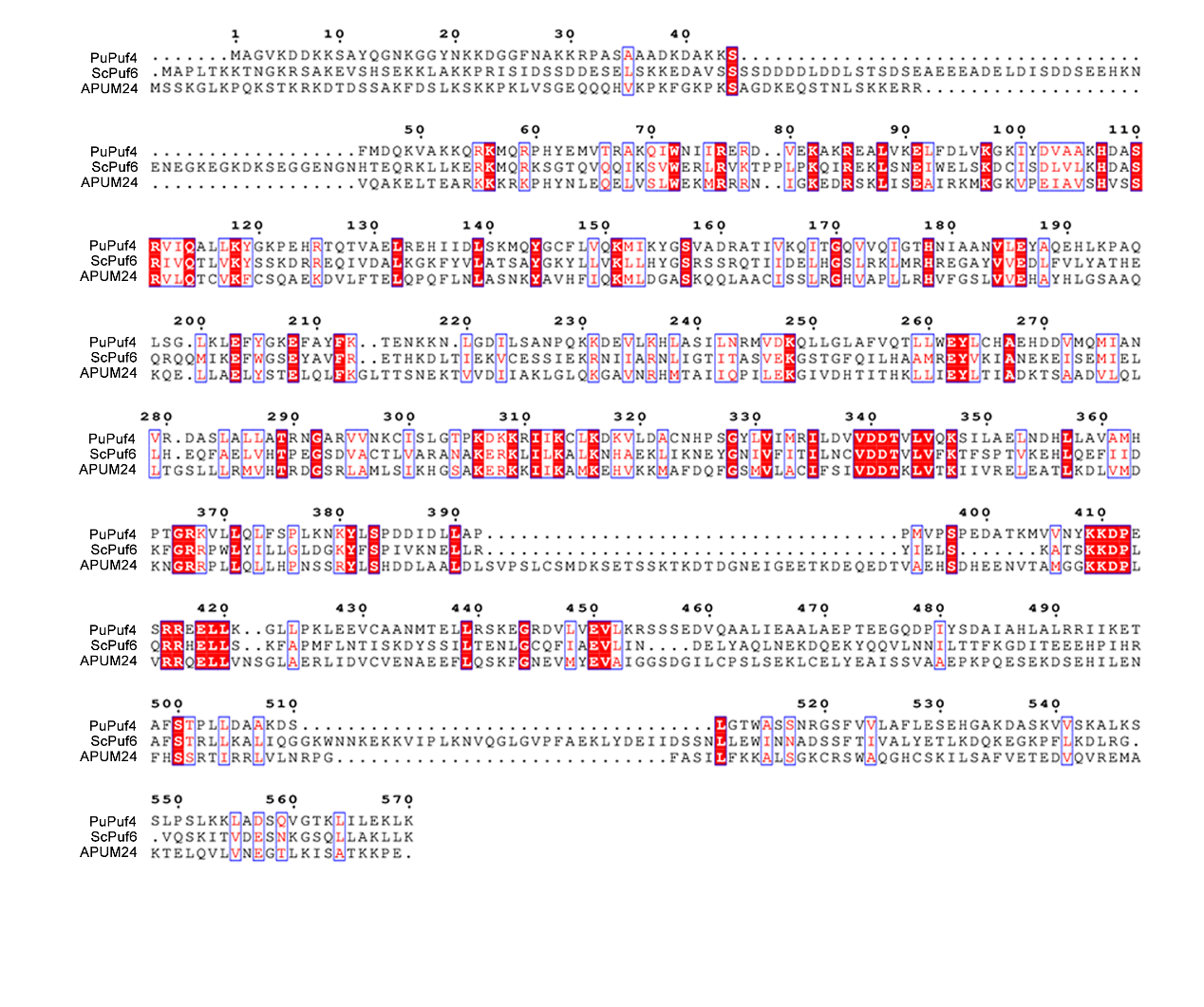
**

**S12 Fig.** **Amino acid alignment of PuPuf4, ScPuf6 and APUM24.**

Supplement: S12 Fig — (DOCX) [file ppat.1013379.s012.docx]
