# Supplementary material for: A non-classical PUF family protein in oomycetes functions as a pre-rRNA processing regulator and a target for RNAi-based disease control
Source: PLoS Pathog. 2025 Jul 31;21(7):e1013379. doi: 10.1371/journal.ppat.1013379 (PMC12324679; doi:10.1371/journal.ppat.1013379)
Supplement: S13 Fig — (A) Schematic diagram of homology-directed repair-mediated modification of the target gene, an ‘all-in-one’ plasmid (pYF515) harboring both Cas9 and sgRNA cassettes was co-transformed with a plasmid (pBS-SK II+) containing homologous donor DNA hph with PsPuf4 flanking sequences. Locations of the primers used to screen the HRR mutants and Sanger sequencing traces of junction regions confirming that the PsPuf4 ORF was precisely replaced. (B) Analysis of genomic DNA from the wildtype (WT), empty-vector control line (EV), and PsPuf4-knockout mutants (ΔPsPuf4-1/2/3) using the primers shown at the top and actin primers as a positive control. (DOCX) [file ppat.1013379.s013.docx]

**
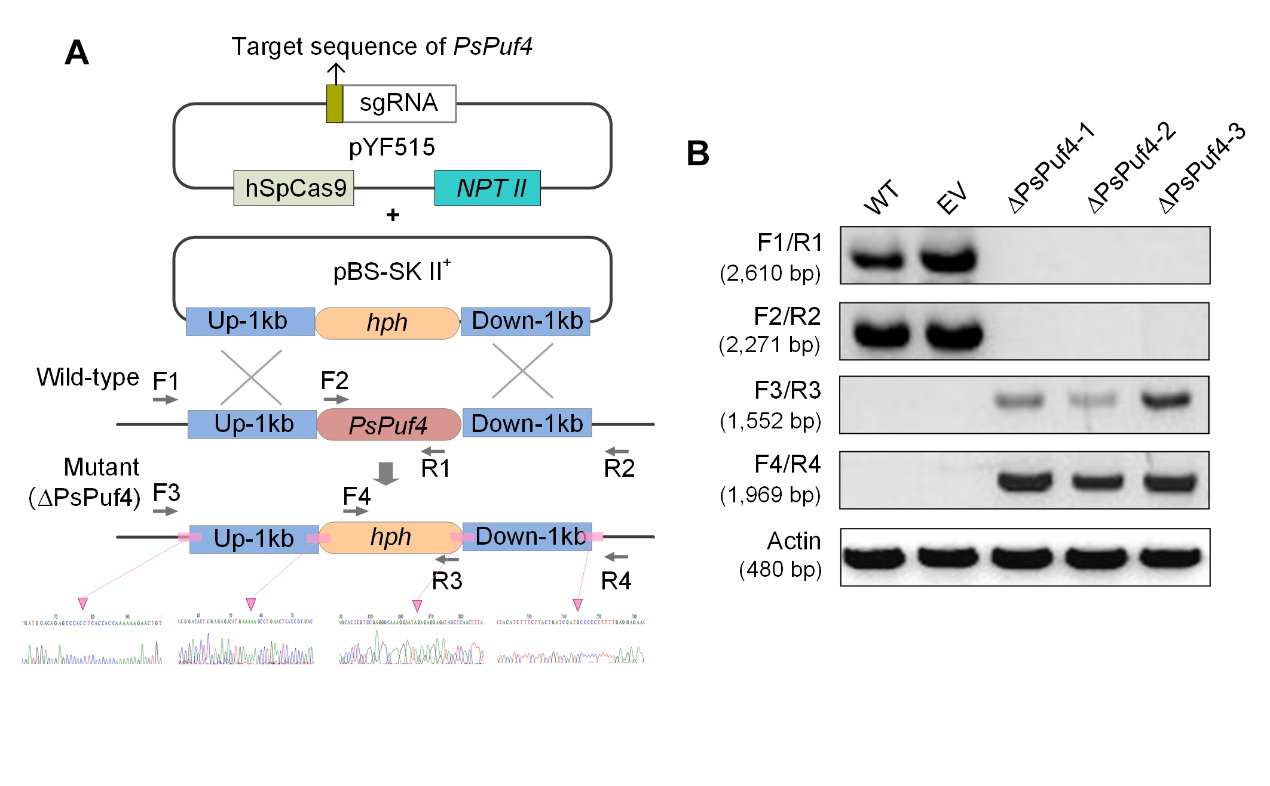
**

**S13 Fig. CRISPR-mediated gene replacement of *PsPuf4*.** (A) Schematic diagram of homology-directed repair-mediated modification of the target gene, an ‘all-in-one’ plasmid (pYF515) harboring both Cas9 and sgRNA cassettes was co-transformed with a plasmid (pBS-SK II+) containing homologous donor DNA *hph* with *PsPuf4* flanking sequences. Locations of the primers used to screen the HRR mutants and Sanger sequencing traces of junction regions confirming that the *PsPuf4* ORF was precisely replaced. (B) Analysis of genomic DNA from the wildtype (WT), empty-vector control line (EV), and *PsPuf4*-knockout mutants (Δ*PsPuf4*-1/2/3) using the primers shown at the top and actin primers as a positive control.
