# Supplementary material for: A non-classical PUF family protein in oomycetes functions as a pre-rRNA processing regulator and a target for RNAi-based disease control
Source: PLoS Pathog. 2025 Jul 31;21(7):e1013379. doi: 10.1371/journal.ppat.1013379 (PMC12324679; doi:10.1371/journal.ppat.1013379)
Supplement: S15 Fig — (A) Oospore formation of WT, EV, ΔPsPuf4 grown on 10% V8 solid medium for 2d, 6d and 14d (Lines one, two and three), Morphology of oospore from 7-day-old cultures grown in V8 liquid medium (The fourth line). Bar, 20 μm. (B) Oospore number cultured for 2d, 6d and 14d. Asterisks indicate significant differences comparing with WT at P < 0.01 (**). (DOCX) [file ppat.1013379.s015.docx]

**
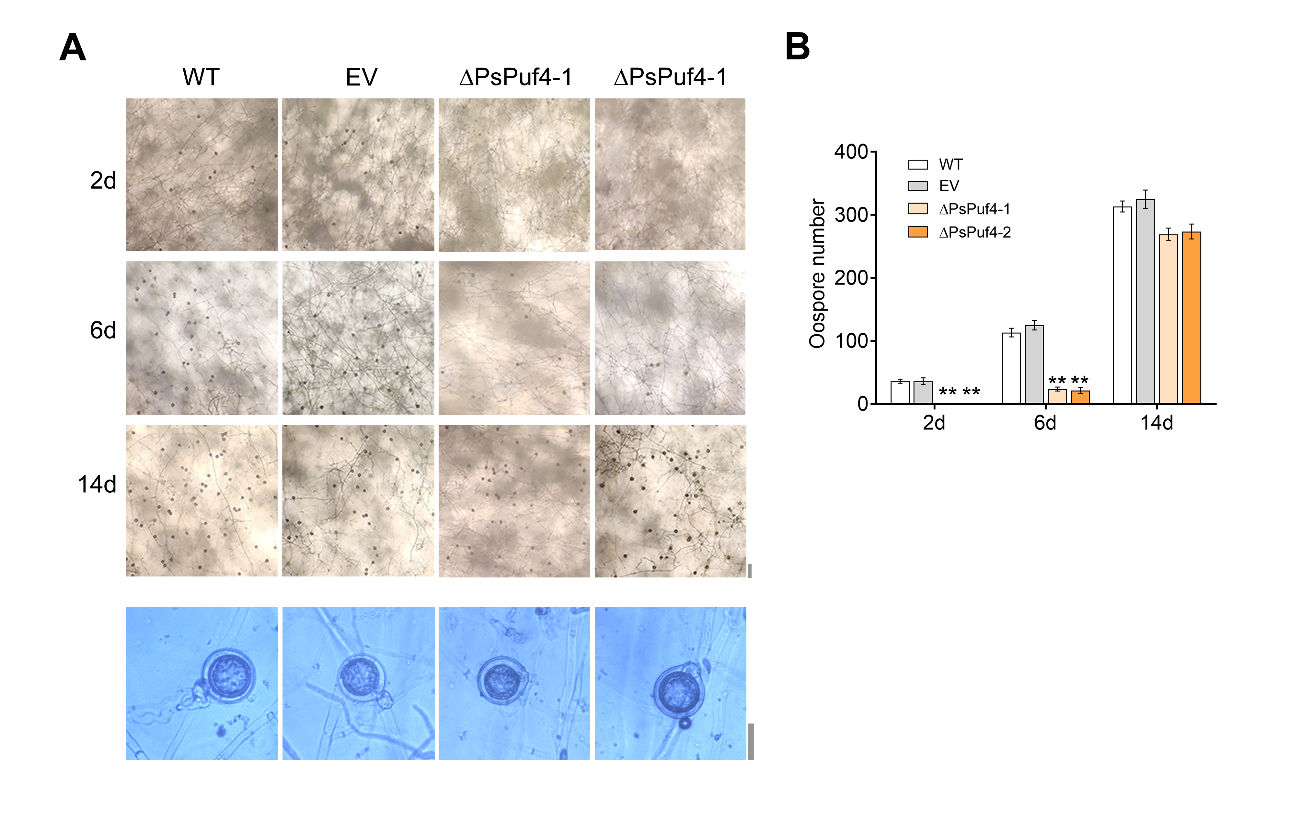
**

**S15 Fig. Oospore formation of WT, EV and ΔPsPuf4 after culture for 2d, 6d and 14d.** (A) Oospore formation of WT, EV, ΔPsPuf4 grown on 10% V8 solid medium for 2d, 6d and 14d (Lines one, two and three), Morphology of oospore from 7-day-old cultures grown in V8 liquid medium (The fourth line). Bar, 20 μm. (B) Oospore number cultured for 2d, 6d and 14d. Asterisks indicate significant differences comparing with WT at P < 0.01 (**).
