# Supplementary material for: A non-classical PUF family protein in oomycetes functions as a pre-rRNA processing regulator and a target for RNAi-based disease control
Source: PLoS Pathog. 2025 Jul 31;21(7):e1013379. doi: 10.1371/journal.ppat.1013379 (PMC12324679; doi:10.1371/journal.ppat.1013379)
Supplement: S16 Fig — (A) Infection lesions on soybean hypocotyl 48 h post-inoculation. (B) Relative Ph. sojae biomass detected through qRT-PCR at 48 h after hypocotyl infection. Asterisks indicate significant differences comparing with WT at P < 0.01 (**). (DOCX) [file ppat.1013379.s016.docx]

**
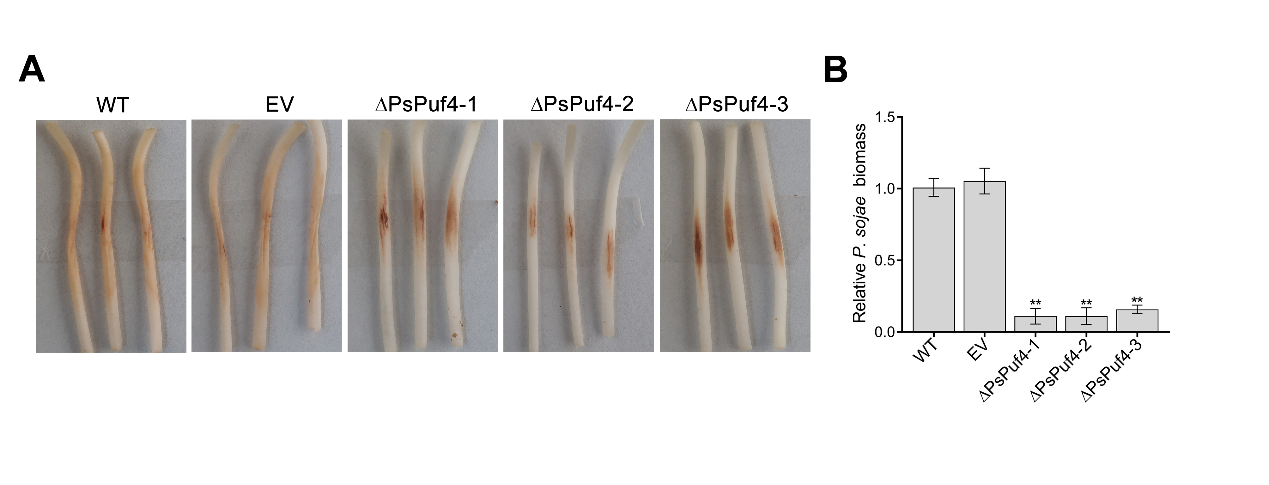
**

**S16 Fig. *PsPuf4* is required for vegetative growth and virulence of *Ph. sojae* and *PsPuf4*-dsRNA compromises the virulence of *Ph. sojae* on soybean.** (A) Infection lesions on soybean hypocotyl 48 h post-inoculation. (B) Relative *Ph. sojae* biomass detected through qRT-PCR at 48 h after hypocotyl infection. Asterisks indicate significant differences comparing with WT at P < 0.01 (**).
