# Supplementary material for: A non-classical PUF family protein in oomycetes functions as a pre-rRNA processing regulator and a target for RNAi-based disease control
Source: PLoS Pathog. 2025 Jul 31;21(7):e1013379. doi: 10.1371/journal.ppat.1013379 (PMC12324679; doi:10.1371/journal.ppat.1013379)
Supplement: S17 Fig — (DOCX) [file ppat.1013379.s017.docx]

**
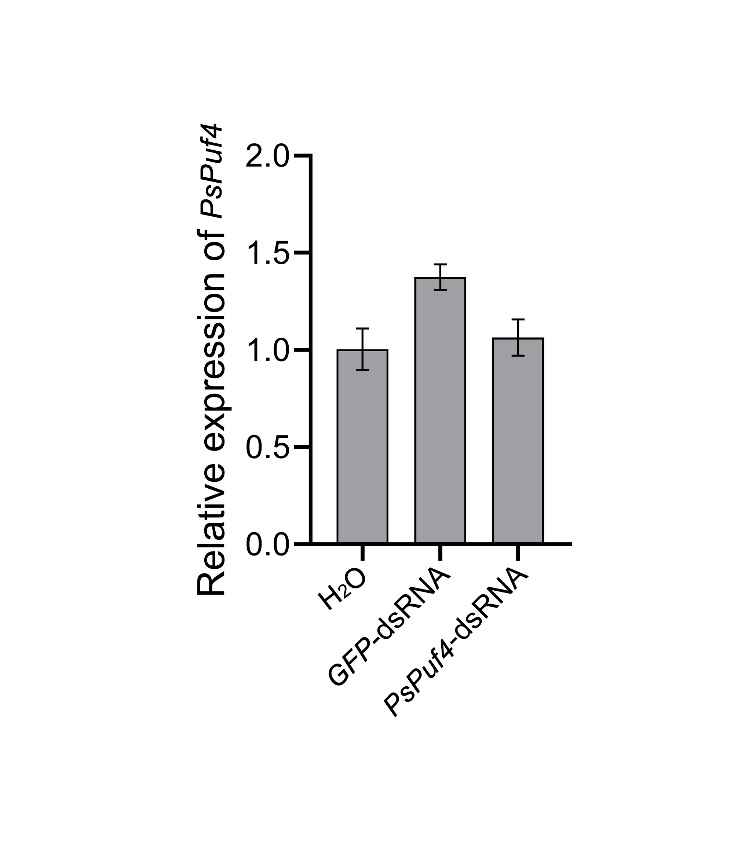
**

**S17 Fig.** **The transcript level of *PsPuf4* in both *PsPuf4*-dsRNA, *GFP*-dsRNA and H_2_O treated zoospore were measured.**
